# Complete Genome Sequence of *Enterobacter roggenkampii* ED5, a Nitrogen Fixing Plant Growth Promoting Endophytic Bacterium With Biocontrol and Stress Tolerance Properties, Isolated From Sugarcane Root

**DOI:** 10.3389/fmicb.2020.580081

**Published:** 2020-09-22

**Authors:** Dao-Jun Guo, Rajesh Kumar Singh, Pratiksha Singh, Dong-Ping Li, Anjney Sharma, Yong-Xiu Xing, Xiu-Peng Song, Li-Tao Yang, Yang-Rui Li

**Affiliations:** ^1^College of Agriculture, Guangxi University, Nanning, China; ^2^Key Laboratory of Sugarcane Biotechnology and Genetic Improvement (Guangxi), Ministry of Agriculture, Sugarcane Research Center, Chinese Academy of Agricultural Sciences, Nanning, China; ^3^Guangxi Key Laboratory of Sugarcane Genetic Improvement, Sugarcane Research Institute, Guangxi Academy of Agricultural Sciences, Nanning, China; ^4^Guangxi Key Laboratory of Crop Genetic Improvement and Biotechnology, Nanning, China; ^5^Microbiology Institute, Guangxi Academy of Agricultural Sciences, Nanning, China

**Keywords:** endophyte, *E. roggenkampii*, nitrogen fixation, PGPB, root colonization, stress, sugarcane, whole-genome sequencing

## Abstract

Sugarcane is the leading economic crop in China, requires huge quantities of nitrogen in the preliminary plant growth stages. However, the use of an enormous amount of nitrogen fertilizer increases the production price, and have detrimental results on the environment, causes severe soil and water pollution. In this study, a total of 175 endophytic strains were obtained from the sugarcane roots, belonging to five different species, i.e., *Saccharum officinarum*, *Saccharum barberi*, *Saccharum robustum*, *Saccharum spontaneum*, and *Saccharum sinense*. Among these, only 23 *Enterobacter* strains were chosen based on nitrogen fixation, PGP traits, hydrolytic enzymes production, and antifungal activities. Also, all selected strains were showed diverse growth range under different stress conditions, i.e., pH (5–10), temperature (20–45°C), and NaCl (7–12%) and 14 strains confirmed positive *nifH*, and 12 strains for *acdS* gene amplification, suggested that these strains could fix nitrogen along with stress tolerance properties. Out of 23 selected strains, *Enterobacter roggenkampii* ED5 was the most potent strain. Hence, this strain was further selected for comprehensive genome analysis, which includes a genome size of 4,702,851 bp and 56.05% of the average G + C content. Genome annotations estimated 4349 protein-coding with 83 tRNA and 25 rRNA genes. The CDSs number allocated to the KEGG, COG, and GO database were 2839, 4028, and 2949. We recognized a total set of genes that are possibly concerned with ACC deaminase activity, siderophores and plant hormones production, nitrogen and phosphate metabolism, symbiosis, root colonization, biofilm formation, sulfur assimilation and metabolism, along with resistance response toward a range of biotic and abiotic stresses. *E. roggenkampii* ED5 strain was also a proficient colonizer in sugarcane (variety GT11) and enhanced growth of sugarcane under the greenhouse. To the best of our knowledge, this is the first information on the whole-genome sequence study of endophytic *E. roggenkampii* ED5 bacterium associated with sugarcane root. And, our findings proposed that identification of predicted genes and metabolic pathways might describe this strain an eco-friendly bioresource to promote sugarcane growth by several mechanisms of actions under multi-stresses.

## Introduction

Agricultural extension in the 20th era has been deeply managed by the application of farm technologies, high-quality varieties, strong tillage, irrigation, chemical fertilizers, and pesticides (Foley et al., 2005). Sugarcane is the main energy and sugar crop that is consumed in several industries as a raw material. China positions the third major sugarcane growing country and produces approximately ten million tons of sugar every year (FAO, 2018) and Guangxi is the leading sugar-producing region of China (Li et al., 2016). The nitrogen fertilizer use is very high for commercial sugarcane production in China, extremely greater as compared to Brazil and other nations (Li et al., 2015). Whereas, constant exploit of nitrogen (N) fertilizers for an extended time increases the production cost as well as causes harmful results on the soil and environment health (Li and Yang, 2015).

Environmentally protected approaches such as bio-fertilizers are seriously required to improve crop/sugarcane growth, nitrogen fixation, and reduce yield loss in different stress conditions to retain sustainable crop production. The utilization of plant growth-promoting (PGP) endophytic bacteria is an efficient approach to stabilizing and improving crop yield due to these bacteria may have ecological benefits more than epiphytic and rhizospheric bacteria as they directly contact with the plants (James, 2000). Endophytic microbes, inhabit and survive inside plant tissue are widely investigated in several plants (Hardoim et al., 2015; Kumar et al., 2017), can support plant growth by several ways such as improving the soil nutrient uptake and germination rate, altering the phytohormone levels and improving plant biotic and abiotic stresses. In addition, secondary aids consist of the biological control of plant pathogens and the induction of induced systemic resistance (ISR) in plants (Rosenblueth and Martínez-Romero, 2006; Ryan et al., 2008; Mei and Flinn, 2010).

Biological nitrogen fixation (BNF) has been confirmed to give 30–80% of the total N for the sugarcane (Boddey et al., 1991; Döbereiner, 1997; Taulé et al., 2012; Urquiaga et al., 2012; Santi et al., 2013). Several nitrogen-fixing bacteria have been reported from inside and rhizosphere of sugarcane plants can fix N related to sugarcane plants (Gillis et al., 1989; Sevilla et al., 2001; Oliveira et al., 2002; Baldani and Baldani, 2005; Li et al., 2017). BNF decreases the sugarcane production cost and sugarcane is cultivated by an extremely less quantity of N inputs as a result of BNF in Brazil (Yong-Xiu et al., 2015). Diazotrophic endophytes are adaptable microorganisms, able to supply nutrients even in absence of nodules in plants, and a method named associative N-fixation (Carvalho et al., 2014). Previous studies showed that a few nitrogen-fixing genera of Enterobacteriaceae family have been enhanced nitrogenase activity and N-fixation in sugarcane (Mirza et al., 2001; Loiret et al., 2004; Govindarajan et al., 2007; Magnani et al., 2010; Lin et al., 2012; Taulé et al., 2012). Hence, there is a need to explore the endophytic diazotrophs belongs to *Enterobacter* genera in the major non- leguminous crops like sugarcane to improve nitrogen fixation, minimize the production cost, decrease the use of chemical fertilizer, and reduce environmental pollution.

Sugarcane production is usually affected by many pathogens and that can accrue in germplasm of sugarcane and cause major crop harm constraining the growth, dropping the stalk weight, and interrupt the sugar recovery. At present, above 120 diseases have been accounted for worldwide (Chen, 1982; Rott et al., 2000), whereas above 60 have been accounted for in China (Lu et al., 1997; Huang and Li, 2014, 2016). Out of these, pokkah-boeng, pineapple, red rot, smut, and wilt diseases cause significant yield damage (Viswanathan and Rao, 2011). Sugarcane production is also influenced by several abiotic stresses like drought, heavy metal, pH, temperature, and salt. NaCl is the major leading salt causing soil salinity, which affects plant growth and yield. Enormous effects of elevated salinity in plants consist of enzyme inactivation; reduction in K and Ca uptake by plants, protein synthesis inhibition, premature leaves senescence, development of burn-like lesions, a decline in respiration, and photosynthesis rate, and loss of cellular integrity, etc. (Munns, 2002). Whereas, heavy metals accretion in soils directly influences the pH and texture of the soil and finally may decrease the plant’s growth by exerting harmful results on a variety of biological processes in plants (Moftah, 2000). Additionally, drought stress stimulates cellular reactive oxygen species (ROS) production, which can oxidize many cellular components, lastly triggering cell death (Barrera, 2012).

The complete-genome study can be used to categorize genes implicated in the positive effects of plant growth-promoting bacteria (PGPB), offer the perception of the molecular and functional mechanisms (Kang et al., 2016; Qin et al., 2017; Oh et al., 2018). Earlier, complete genome analysis of some other *Enterobacter* stains is accessible (Ren et al., 2010; Taghavi et al., 2010; Liu et al., 2012; Andrés-Barrao et al., 2017) excluding *E. roggenkampii*. Therefore, the complete genome sequence accessibility of endophytic *E. roggenkampii* isolated from sugarcane root will help in full understanding of the diverse biological mechanisms and determining the characteristics of this bacteria, plus gene identification that is contributing to the positive activity of PGPB, improve sugarcane growth under abiotic and biotic stresses.

The objectives of this research are (i) to isolate *Enterobacter* strains from the roots of five different sugarcane species grown in the field of Guangxi, China (ii) to study their plant growth-promoting (PGP) and nitrogenase activities, as well as biocontrol potential against sugarcane and other plant pathogens (iii) to detect the *nifH* and *acdS* genes amplification (iv) to study their hydrolytic enzymes (chitinase, glucanase, cellulase, and protease) production (v) to investigate their capacity to tolerate several abiotic stresses (pH, temperature, and NaCl), (vi) to examine the colonization pattern of selected most prominent *E. roggenkampii* ED5 strain in sugarcane plant through confocal laser scanning microscopy (CLSM) and scanning electron microscopy (SEM), and (vii) to sequence the *E. roggenkampii* ED5 genome, a prospect to create the allocation of nitrogen-fixing, PGP, and stress-related genes. Here, we report the first statement of the forthcoming application of *E. roggenkampii* ED5 endophytic bacteria, isolated from sugarcane root, as a potential agent to improve growth and nitrogen fixation in sugarcane, stress alleviation, and biocontrol against pathogens.

## Materials and Methods

### Sugarcane Samples Collection

Five different sugarcane species were selected in this study, i.e., *Saccharum officinarum*, *Saccharum barberi*, *Saccharum robustum*, *Saccharum spontaneum*, and *Saccharum sinense*. All these five sugarcane plant samples were obtained from the nursery of Sugarcane Germplasm Resources, Guangxi Academy of Agricultural Sciences, Sugarcane Research Centre, Nanning, China. Only root samples were selected for the isolation of different endophytic bacteria at the elongation stage. For each sample, five plants were selected, and five different root samples were composed of each sugarcane species. The roots samples with white tips, indicated the active growth, were used for the isolation.

### Isolation and Cultivation of the Strain

One gram of fresh roots pieces was squashed in one mL of sterile 5% sucrose solution after sterilization (Dobereiner et al., 1993). Roots were cleaned with tap water, disinfected superficially by 70% ethanol for 5 min, once more rinsed and disinfected with 3% sodium hypochlorite for 5 min. For sterilization, roots were cleaned by sterilized double-distilled water, and then samples were dried with sterile filter paper. To check the disinfection method accomplishment, the former washing double distilled water was spread on the nutrient agar (NA) and potato dextrose agar (PDA) plates, kept at 30 ± 2 and 26 ± 2°C in an incubator for 3–5 days. The results were utilized as a sterilization control, and no fungal and bacterial colonies were capable to develop on the plates (Slama et al., 2019). Six different media were chosen for the nitrogen-fixing endophytic bacteria isolation, i.e., Ashby’s glucose, Ashby’s mannitol, Burk medium, Jensen medium, NA, and Yeast mannitol agar. The composition of all used different media is provided in Supplementary Table S1. All root samples were crushed with 5% of the sucrose solution. And, ten-fold serial dilutions from 10^–2^ to 10^–5^ of the 100 μL aliquots suspensions were spread into all different mediums in triplicates. After morphologically different strains of emerging spots or layers from the root, pieces were selected after 5–7 days at 30 ± 2°C, and individually bacterial colonies were further successive purification. The endophytic bacterial strains were stored in 25% glycerol at −20°C.

### Antagonism Assay Against Phytopathogenic Fungi

All endophytic strains were assessed for their *in vitro* antifungal activities against *Fusarium moniliforme*, *Fusarium cubense*, *Botrytis cinereal*, *Ceratocystis paradoxa*, and *Sporisorium scitamineum* with the slight modification of Singh et al. (2020) method on NA plus PDA (1:1) medium. These all fungal pathogens were obtained from Agriculture College, Guangxi University, Nanning. A 5 mm diameter of actively growing pathogen culture disk was cut from the PDA plate and put in the middle of PDA: NA plates. All bacterial strains (10^6^ cell mL^–1^) were streaked on the plate around 3 cm from the pathogen disk and kept at 28 ± 2°C, till the mycelia of fungal pathogens were completely grown in the control plate (without bacterial strains). The antifungal activity was evaluated by determining the growth inhibition in response to selected pathogens. The inhibition percentage was observed by Singh et al. (2013) and strains displaying ≥50% inhibition of mycelial growth were measured as potential biocontrol agents.

### Estimation of Cell Wall Degrading Enzymes Activity

The hydrolytic enzymes production is a common mechanism used by bacteria to prevent the growth of pathogenic microorganisms. In this study, production of four hydrolytic enzymes, i.e., chitinase (catalog no. MM1062O1), protease (MM1206O1), glucanase (MM91504O1), and cellulase (MM91502O1) was measured by enzyme-linked immune sorbent assays (ELISA) kits (Wuhan Colorful Gene Biological Technology Co. Ltd, China). A pure colony was transferred into 10 mL of LB broth medium and placed at 180 rpm for 36 h at 32°C in incubator shaker. Bacterial culture was centrifuged at 12,000 rpm for 5 min to acquire a supernatant. The supernatant for all strains was used for different enzyme activities assay by ELISA kits. The complete extraction method was performed at 4°C. The ELISA was done in 96-well microtiter plates coated with the antigen against the selected enzymes, according to Singh et al. (2018); Singh P. et al. (2019) procedure.

### *In vitro* Screening of Endophytic Isolates for Abiotic Stress Tolerance

Growth of all selected endophytic bacterial strains was examined for their capacity to tolerate several abiotic stress conditions, i.e., temperature (20–45°C), pH (5–10), and NaCl (7–12%) in LB broth by spectrophotometer at 600 nm and the uninoculated medium was used as a blank.

#### Temperature Tolerance

0.1 mL bacterial suspension was transferred in LB broth medium (5 mL) of and tubes were incubated at 20, 25, 30, 35, 40, and 45°C for 36 h in a shaker incubator at 120 rpm and O.D. was recorded at 600 nm.

#### pH Tolerance

The pH of the LB broth medium was attuned to 5, 6, 7, 8, 9, and 10 with sterile buffers. 0.1 mL fresh cultures were transferred in 5 ml of LB broth medium comprising different pH and kept at 37°C; 120 rpm in incubator shaker and after 36 h growth was measured at 600 nm.

#### Salinity Tolerance

Five mL of LB broth medium supplemented with 7, 8, 9, 10, 11, and 12% NaCl was distributed in 30 mL tubes and autoclaved. 0.1 mL bacterial suspension was inoculated in LB broth tubes and incubated at 37°C/120 rpm in shaker incubator and growth was calculated at 600 nm after 36 h.

### Screening for PGP Activities

All endophytic strains were examined for different PGP traits, i.e., Indole acetic acid (IAA), Phosphate (P) solubilization, siderophore, hydrogen cyanide (HCN), and ammonia production, following the standard protocol of Lorck (1948), Schwyn and Neilands (1987), Brick et al. (1991), Glickmann and Dessaux (1995), and Dey et al. (2004), respectively. Each analysis was completed in three biological repeats.

Indole acetic acid production was estimated by the colorimetric method in the presence of tryptophan in the medium at different concentration levels. The potential of bacterial isolates to solubilize P was qualitatively evaluated by the Pikovskaya medium supplemented with tri-calcium phosphate. The strains were transferred on a plate and kept at 30 ± 2°C for 5–7 days and the development of a clear hallow zone around the bacterial isolates indicated P-solubilizing capacity. All selected endophytic strains were screened for siderophores production and development of halo zone on the chrome azurol S medium confirmed siderophore production. The HCN production capacity of all strains was evaluated on PDA medium with 4.4 g L^–1^ glycine to produce hydrocyanic acid. A filter paper soddens with 0.5% picric acid and 2% Na_2_CO_3_ was put on a cover plate, after that sealed by Parafilm and kept at 28°C, and change in color of filter paper confirmed the HCN production. All strains were incubated in 10% sterile peptone H_2_O at 30 ± 2°C for 72 h and change in yellow color by the addition of Nessler’s reagent (0.5 mL) confirmed the ammonia production.

### Determination of 1-Aminocyclopropane-1-Carboxylate (ACC) Deaminase Assay

1-Aminocyclopropane-1-carboxylate deaminase activity of all strains was studied based on the capability to utilize ACC as a nitrogen source on nitrogen-free Dworkin and Foster (DF) medium (Jacobson et al., 1994). DF medium deprived of ACC was used as the negative control, whereas DF medium with ACC (3 mM) or (NH_4_)_2_SO_4_ (0.2% w/v) was used as a positive control. The plates were kept at 30 ± 2°C for 3–5 days and ACC deaminase activity was confirmed by the strain growth on ACC plates. Quantitative ACC deaminase activity estimation was estimated by the procedure of Honma and Shimomura (1978).

### Acetylene Reduction Assay (ARA)

The nitrogen-fixing capacity of each strain was examined by the ARA method (Hardy et al., 1968), and the procedure was followed by Li et al. (2017) with some modification.

### Molecular Characterization and Phylogenetic Analysis

Genomic DNA was isolated for all selected endophytic strains with DNA isolation kit (CWBIO, Beijing, China) and DNA was confirmed by gel electrophoresis (0.8% w/v) and quantified by Nanophotometer spectrophotometer (Pearl, Implen-3780). The 16S rRNA gene was amplified by using a pair of pA-F and pH-R universal primer through PCR and PCR condition was followed as Li et al. (2017) (Supplementary Table S2), and the purified PCR product was sequenced (Sangon Biotech, Shanghai, China).

Phylogenetic analysis and evolutionary relationship of the selected *Enterobacter* strains were studied through the comparison of 16S rRNA gene sequences with reference sequences of the National Center for Biotechnology Information (NCBI) GenBank database. The alignment of sequences was completed with ClustalW (Saitou and Nei, 1987). The phylogenetic tree was created by molecular evolutionary genetics analysis (MEGA) software (version 7.0) (Kumar et al., 2016) and unweighted pair group process through arithmetic mean (UPGMA) (Sneath and Sokal, 1973) in a Kimura two-parameter model (Tamura et al., 2004). The bootstrap examination was finished via Felsenstein procedure with 1000 pseudoreplication (Felsenstein, 1985).

### *nifH* and *acdS* Genes Amplification

The *nifH* and *acdS* genes amplification of all the selected strains was achieved with degenerate sets of primer following the PCR conditions of Li et al. (2011, 2017), as presented in Supplementary Table S2. All amplified products of PCR were purified and cloned according to the manufacturer’s instructions (TaKaRa, Japan) and then sequenced (Sangon Biotech, Shanghai, China). All sequences obtained for both genes were checked through the blastn suite search engine in the NCBI GenBank database.

### Root Colonization Study of *E. roggenkampii* ED5

The root colonization inside the sugarcane plant was confirmed through Green Fluorescent Protein (GFP) and Scanning Electron Microscopy (SEM) techniques. The pPROBE-pTetr-TT plasmid having the GFP gene was obtained from the Agriculture College, Guangxi University, Sugarcane laboratory, Nanning, China. Strain ED5 was mixed with plasmid vector (1:2 ratio) in LB broth and incubated at 30 ± 2°C for 36 h in an orbital shaker for 120 rpm. Sugarcane plantlets were shifted in the glass bottle inside the bacterial suspension and kept in the growth chamber. After 72 h plantlets were taken away and washed with autoclaved water. Micro-propagated cultivated sugarcane plantlets were cut into a small section and observed by a CLSM (Leica DMI 6000, Germany) (Singh et al., 2020). Sugarcane plant samples (stem and root tissues) were selected for the SEM analysis, both samples were cut into small pieces by knife and fixed in glutaraldehyde solution (Catalog G1102, Servicebio) overnight at 4°C. The samples were washed three times with distilled water and dehydrated in ethanol 30, 50, 70, 90, 95, and 100% for 15 min and finally isoamyl acetate for 15 min. After drying the samples with critical point dryer, colonization of *E. roggenkampii* ED5 was observed in sugarcane by using the SEM (Hitachi model SU8100), according to the protocol of Singh et al. (2013).

### Evaluation of Plant Growth Parameters

The different plant growth parameters such as chlorophyll content, leaf area, plant height, root weight, shoot weight, photosynthesis, and transpiration rate were observed in sugarcane variety GT11 at 30 and 60 days after inoculation of strain ED5.

### DNA Extraction, Library Construction, and Genome Sequencing

Genomic DNA was isolated from the overnight liquid cell suspension of *E. roggenkampii* strain by Wizard Genomic DNA Kit (Promega). DNA quality and concentration were estimated by TBS-380 fluorometer (Turner BioSystems Inc., Sunnyvale, CA, United States) and DNA with high quality (OD_260_/_280_ = 1.8 ∼ 2.0 > 20 μg) was employed for additional experiment. The genome was sequenced by a fusion of Nanopore and Illumina sequencing platforms. The Illumina data were employed to assess the complexity of the genome. For Illumina sequencing, as a minimum 1 μg genomic DNA was utilized for every isolate in the assembly of the sequencing library. DNA fragments were incised into 400–500 bp by a Covaris M220 Focused Acoustic Shearer. Illumina sequencing libraries were prepared by NEXTflex Rapid DNA-Seq Kit. Briefly, 5′ prime ends were first end-repaired and phosphorylated. Next, the 3′ ends were A- tailed and ligated to sequencing adapters. The third step was to enrich adapters-ligated products using PCR. The organized libraries were used for paired-end Illumina sequencing (2 × 150 bp) on an Illumina HiSeq X Ten. For Nanopore sequencing, 15 μg of genomic DNA was spin in a Covaris G-TUBE (Covaris, MA) to cut the genomic DNA into ∼10 kb fragments, then performed magnetic bead purification and connect the sequencing adapters to both ends.

### Genome Assembly, Annotation, and Gene Prediction

The data obtained by Nanopore and Illumina platform were used for bioinformatics analysis and all the analyses were done with the free online platform of Majorbio Cloud Platform^1^ from Shanghai Majorbio Bio-pharm Technology Co., Ltd. The whole-genome sequence was assembled using both Nanopore reads and Illumina reads. A statistic of quality information was applied for quality trimming, by which the low-quality data can be removed to form clean data. The reads then assembled into a contig using a hierarchical genome assembly process (HGAP) and canu (Koren et al., 2017), and the circular step was checked and completed, generating a complete genome with seamless chromosomes and plasmids. Finally, error correction of the Nanopore assembly results was performed using the Illumina reads using Pilon.

The Glimmer version 3.02 was used for coding sequence (CDS) prediction and predicted CDSs were annotated from NR, Swiss-Prot, Pfam, Gene Ontology (GO), Clusters of Orthologus Groups (COG) and Kyoto Encyclopedia of Genes and Genomes (KEGG) databases (Delcher et al., 2007) by sequence alignment tools, i.e., *Basic Local Alignment Search Tool* (BLAST), Diamond and HMMER. The tRNA-scan-SE (v1.2.1) (Borodovsky and Mcininch, 1993) and Barrnap were used for tRNA prediction and rRNA prediction, as well as antismash software was used for the secondary metabolite genes prediction. In short, every protein query was aligned, and annotations of accurately matched subjects (*e*-value < 10^–5^) were completed for gene annotation.

### Phylogenetic Analysis Based on Average Nucleotide Identity (ANI) Calculations

Complete genome similarity was calculated with ANI. The *Enterobacter* strains gene sequences were obtained from the NCBI database. Based on the selected *E. roggenkampii* ED5 16S rRNA gene and 10 house-keeping genes (*dnaG*, *frr*, *rpoB*, *pgk*, *rplB*, *infC*, *pyrG*, *rpmA*, *smpB*, and *rpsB*) online NCBI Blast search program^2^ was used to compare the ED5 strain with closely related eight strains. ANI results were analyzed using R version 3.5.1 gplots 3.0.4 software and presented as heat map and vegan 2.5–6 software was used for hierarchical cluster analysis.

### Statistical Analysis

All genome analysis process was completed by the manufacturer’s instructions. All PGP and biocontrol tests were done in three replicates and data were considered through analysis of variance followed by Duncan’s multiple range test. Data were showed as the mean plus the standard error of the mean and evaluated by the Student *t*-test with *p*-value < 0.05 was indicated significant.

## Results

### Isolation and PGP Activities of Endophytic Bacteria From Sugarcane Roots

A total of 175 endophytic bacterial strains were isolated by using six different selective mediums from the roots of five sugarcane species (*S. officinarum*, *S. barberi*, *S. robustum*, *S. spontaneum*, and *S. sinense*). Among these, only 90 strains were selected which exhibited various nitrogenase and PGP activities, as well as biocontrol potential against sugarcane and other crops pathogens. After 16S rRNA gene sequencing, we preferred only 23 Enterobacter strains for further study (Supplementary Figure S1). PGP activities of all 23 selected strains are presented in Table 1.

**TABLE 1:** Plant growth-promoting (PGP) and biocontrol activities of selected endophytic strains from the roots of different sugarcane species.

Isolates	PGP-traits					Biocontrol activity/				
	Siderophore	Phosphate	ACC	HCN	Ammonia	*F. moniliforme*	*F. cubense*	*B. cinerea*	*C. paradoxa*	*S. scitamineum*
AA1	−	++	+	−	+++	+	−	−	+	−
AH1	−	+	+	++	+++	−	+++	++	−	+
BC1	++	++	++	++	++	+++	+++	+++	+++	+++
BC2	++	+++	+	−	−	−	+	+++	−	+++
BD1	++	−	+	−	+++	−	+	−	−	+++
CA1	++	+	++	−	++	−	+++	+++	−	++
C10	−	−	+	−	−	−	−	+++	+	−
CI1	++	−	+	−	+++	−	+++	+++	+	+
DF1	−	−	+	++	+++	+++	+++	+++	+++	+++
DH1	−	+	+	++	+++	+++	+++	+++	+++	+++
EB3	+++	+	+	++	−	++	−	−	−	+++
EC5	−	++	+	−	+++	+++	+	−	+	+++
ED4	−	++	+	−	+++	+++	+	++	−	+++
ED5	+++	++	++	++	+++	+++	+++	+++	+++	+++
EF2	−	++	+	++	+++	++	++	++	+	+
EI1	++	+++	+	−	−	++	++	++	+	+
R15	−	−	++	++	+++	−	++	−	+	+
R16	−	++	+	−	+++	+++	+	+++	−	+
AS3	++	++	++	−	−	++	++	++	−	+
AS5	++	−	+	+	+++	++	++	+++	−	+
ACA7	++	++	+		+++	+++	++	++	−	++
ACD1	++	+++	++	++	++	+++	++	+++	−	++
ACD2	++	−	+	−	++	++	++	+++	−	+

*+, low activity; ++, moderate activity; +++, strong activity; –, no activity.*

Out of all 23 strains, *in vitro* siderophore production results showed that 13 (56.52%) strains confirmed positive response by producing halo orange zone in CAS agar medium and two strains (EB3 and ED5) were showed strong activity. For P- solubilization, only 16 (69.56%) strains have the potential to produce a zone of inhibition to solubilize tricalcium phosphate on Pikovskaya’s media and three strains (BC2, EI1, and ACD1) displayed strong activity (Table 1). Both assays were performed by measuring 3 mm or larger zone of inhibition on specific medium following incubation at 30 ± 2°C for 3–5 days. Further, Table 1 indicated that 10 (43.47%) and 18 (78.26%) strains were proficient for HCN, and ammonia production with more strains established positive ammonia production test than that of HCN.

Biocontrol activity of all these endophytic bacteria was also analyzed in response to five different plant pathogens. The results presented in Table 1, designated that 21 (91.3%), 11 (47.82%), 16 (69.56%), 20 (86.95%), and 18 (78.26%) isolates were antagonistic against *S. scitamineum*, *C. paradoxa*, *F. moniliforme*, *F. cubense*, and *B. cinerea* correspondingly, with ED5, DH1, and DF1 strains possessed strong biocontrol activity against all pathogens.

ACCD activity was measured by all the strains which showed the potential to use ACC as a solitary source of nitrogen in DF minimal medium and the result illustrated the growth of all strains on plate medium. In addition, further screened for quantitative ACCD activity and varying ranged of activity was observed by all strains from 212.73 to 1192.74 nmol α-ketobutyrate mg^–1^h^–1^. The highest ACCD activity was examined by strain EB3 followed by ED5 and ED4 (Table 2). The nitrogen-fixing capacity of all isolates was measured through the ARA method which varied from 8.23 to 29.60 nmoL C_2_H_4_ mg protein h^–1^. Strain ED5 recorded the maximum, whereas BC1 showed the minimum nitrogenase activity (Table 2).

**TABLE 2:** *In vitro* quantitative assays for ARA, ACC, and hydrolytic enzymes of isolated endophytic strains.

Isolates	IAA (μg mL^–^^1^)			ARA (nmoL C_2_H_4_ mg protein h^–^^1^)	ACC (nmol α-ketobutyrate mg^–1^ h^–1^)	Hydrolytic Enzymes (IU mL^–1^)			
	AT	PT (0.5%)	PT (1%)			Cellulase	Chitinase	Endoglucanase	Protease
AA1	65.23^*d*^	167.98 ^*j*^	147.71^*k*^	9.91^*k*^	508.36^*j*^	123.34^*jkl*^	183.24^*j*^	529.59^*l*^	154.42^*fg*^
AH1	140.08^*a*^	627.97^*cd*^	159.95^*j*^	14.73^*f*^	509.05^*j*^	125.87^*ijk*^	190.52^*i*^	619.77^*j*^	157.73^*ef*^
BC1	28.30^*i*^	155.34^*k*^	272.74^*g*^	8.23^*m*^	837.90^*e*^	134.75^*h*^	225.51^*f*^	691.93^*h*^	151.77^*ghi*^
BC2	38.13^*f*^	656.27^*b*^	527.42^*a*^	10.54^*j*^	405.46^*m*^	128.40^*ij*^	174.32^*k*^	583.69^*k*^	146.49^*ij*^
BD1	39.34^*f*^	418.85^*f*^	402.59^*f*^	11.17^*i*^	250.40^*q*^	129.66^*i*^	174.51^*k*^	574.67^*k*^	152.44^*fgh*^
CA1	30.71^*h*^	128.04^*m*^	131.25^*lm*^	13.26^*g*^	717.31^*g*^	211.52^*b*^	202.18^*h*^	858.89^*e*^	168.34^*ab*^
C10	26.49^*jk*^	116.00^*n*^	235.01^*h*^	15.77^*e*^	424.53^*m*^	104.63^*m*^	151.24^*m*^	777.65^*g*^	154.42^*fg*^
CI1	20.87^*m*^	76.46^*q*^	155.14^*jk*^	12.21^*h*^	212.73^*r*^	122.08^*kl*^	151.44^*m*^	658.68^*i*^	148.47^*hij*^
DF1	24.69^*l*^	160.15^*jk*^	76.66^*p*^	13.05^*g*^	688.43^*h*^	118.32^*l*^	168.69^*k*^	700.95^*h*^	149.79^*g*–*j*^
DH1	110.78^*c*^	213.94^*i*^	46.16^*q*^	9.07^*l*^	758.85^*f*^	147.59^*g*^	243.03^*e*^	687.42^*h*^	151.77^*ghi*^
EB3	33.11^*g*^	617.93^*d*^	421.26^*e*^	9.28^*l*^	1192.74^*a*^	122.08^*kl*^	170.14^*k*^	786.68^*g*^	157.73^*ef*^
EC5	31.51^*gh*^	635.40^*c*^	435.91^*d*^	13.26^*g*^	657.94^*i*^	193.83^*c*^	231.35^*f*^	849.86^*ef*^	169.01^*ab*^
ED4	47.97^*e*^	142.89^*l*^	139.08^*l*^	8.25^*m*^	966.23^*c*^	127.13^*ijk*^	216.76^*g*^	601.73^*jk*^	153.10^*fgh*^
ED5	123.23^*b*^	732.93^*a*^	517.19^*b*^	29.60^*a*^	1096.10^*b*^	179.07^*d*^	295.68^*b*^	1355.87^*d*^	169.67^*ab*^
EF2	27.29^*ij*^	91.32^*op*^	92.32^*o*^	15.36^*e*^	683.19^*h*^	152.77^*f*^	229.89^*f*^	691.93^*h*^	145.83^*j*^
EI1	31.11^*h*^	524.01^*e*^	471.83^*c*^	10.54^*j*^	881.38^*d*^	93.58^*n*^	269.34^*d*^	592.71^*jk*^	144.51^*j*^
R15	16.86^*p*^	70.64^*q*^	48.97^*q*^	16.40^*d*^	424.28^*m*^	143.72^*g*^	338.20^*a*^	714.49^*h*^	163.03^*cd*^
R16	17.66^*op*^	96.33^*o*^	84.69^*op*^	20.38^*b*^	263.76^*pq*^	107.10^*m*^	278.12^*c*^	696.44^*h*^	148.47^*hij*^
AS3	33.11^*g*^	235.61^*h*^	200.89^*i*^	16.54^*d*^	296.61^*o*^	173.76^*e*^	161.41^*l*^	822.78^*f*^	172.99^*a*^
AS5	19.07^*no*^	534.85^*e*^	147.71^*k*^	20.17^*b*^	454.09^*l*^	156.67^*f*^	155.60^*lm*^	1342.30^*d*^	167.01^*bc*^
ACA7	19.87^*mn*^	96.53^*o*^	128.44^*m*^	15.72^*e*^	273.74^*p*^	146.29^*g*^	136.71^*n*^	1446.33^*b*^	172.99^*a*^
ACD1	25.49^*kl*^	332.55^*g*^	153.53^*jk*^	18.92^*c*^	473.64^*k*^	449.25^*a*^	125.09^*o*^	1554.92^*a*^	167.68^*abc*^
ACD2	11.24^*q*^	81.08^*pq*^	107.57^*n*^	14.52^*f*^	372.76^*n*^	104.63^*m*^	132.35^*n*^	1410.14^*c*^	161.04^*de*^
SEM	0.573	4.000	2.864	0.159	6.806	1.774	2.203	9.572	1.677
CD (*P* = 0.05)	1.632	11.387	8.153	0.453	19.373	5.051	6.272	27.248	4.775
CV (%)	2.400	2.300	2.200	1.900	2.000	2.000	1.900	2.000	1.800

*Means of the similar alphabet within a row are not significantly different (P ≤ 0.05) according to Duncan’s Multiple Range Test (DMRT). SEM, standard error of the difference between means; CD, critical difference; CV, coefficient of variation. AT, absence of tryptophan and PT, the presence of tryptophan*.

### IAA Production

Indole acetic acid biosynthesis is an essential trait of PGPR strains and results elucidated that all these isolates had a diverse ability to synthesize IAA, which are presented in Table 2. The quantitative IAA synthesis ranged from 70.64 to 732.93 μg mL^–1^ and 46.16 to 527.42 μg mL^–1^ in medium supplemented with 0.5 and 1% tryptophan and from 11.24 to 140.08 μg mL^–1^ in medium deprived of tryptophan. In the presence of 0.5% tryptophan, the minimum and maximum IAA production were recorded R15 and ED5 strains. While strains BC2 and DH1 confirmed the highest and lowest IAA production in medium supplemented with 1% of tryptophan. For medium devoid of tryptophan, the greatest IAA production was observed in AH1 and the least for ACD2 strains, respectively.

### Hydrolytic Enzymes Assay

The quantitative estimation of four hydrolytic enzymes, i.e., cellulase, chitinase, endoglucanase, and protease was also measured for all the selected strains using the ELISA kit. All strains showed activity ranged between 93.58–449.25, 125.09–338.2, 529.59–1554.92, and 144.51–172.99 IU mL^–1^ for cellulase, chitinase, endoglucanase, and protease enzymes, respectively (Table 2). The strains R15 and AS3 showed maximum chitinase and protease activities; with ACD1 strain confirmed maximum cellulase and endoglucanase activities. Whereas, ACD1 and AA1 strains presented minimum chitinase and endoglucanase activities, and EI1 strain displayed minimum cellulase and protease activities (Table 2).

### Different Abiotic Stress Tolerance

**FIGURE 1:**
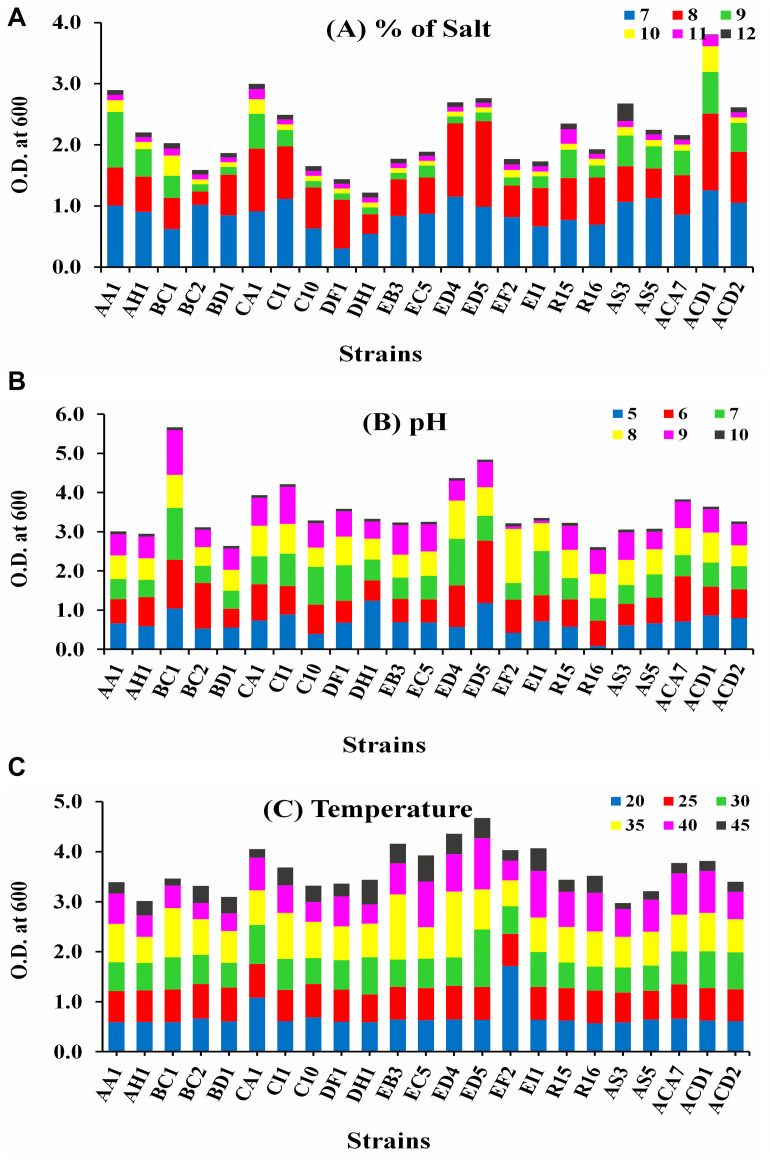
Intrinsic result of various abiotic stresses of selected endophytic *Enterobacter* strains growth **(A)** salt (7–12%), **(B)** pH (5–10), and **(C)** temperature (20–45°C).

The growth of all selected strains was measured at 600 nm in different abiotic stress conditions, i.e., temperature (20–45°C), pH (5–10), and salt (7–12%), as displayed in Figure 1. Strain ACD1 established the greatest growth followed by CA1, AA1, and ED5 strains in LB broth medium supplemented with 7–12% NaCl, whereas the lowest growth was observed by the DH1 strain (Figure 1A). For pH, strains BC1, ED5, ED4, and CI1 showed maximum ability to grow in an extensive pH varying from 5 to 10. Alternatively, stains R16 and BD1 were least pH tolerant (Figure 1B). In the case of temperature, strain ED5 exhibited the highest and AS3 confirmed lowest temperature tolerance up to 45°C (Figure 1C).

### Molecular Classification and Phylogenetic Study of Endophytic Isolates

Endophytic strains were recognized through 16S rRNA gene sequencing and all achieved sequences were matched with nucleotide sequences of the national center for biotechnology information (NCBI) GenBank database by basic local alignment search tool (BlastN) program. We alienated 23 strains into 10 different species of *Enterobacter* i.e., *Enterobacter ludwigii* (2), *Enterobacter cloacae* (5), *Enterobacter tabaci* (2), *Enterobacter* sp. (5), *Enterobacter asburiae* (3), *Enterobacter cancerogenus* (1), *Enterobacter oryzae* (2), *Enterobacter aerogenes* (1), *Enterobacter roggenkampii* (1), and *Enterobacter mori* (1), based on ≥97% score similarity value. And all sequences were deposited in the NCBI GenBank from accession numbers MT613360-MT613382.

**FIGURE 2:**
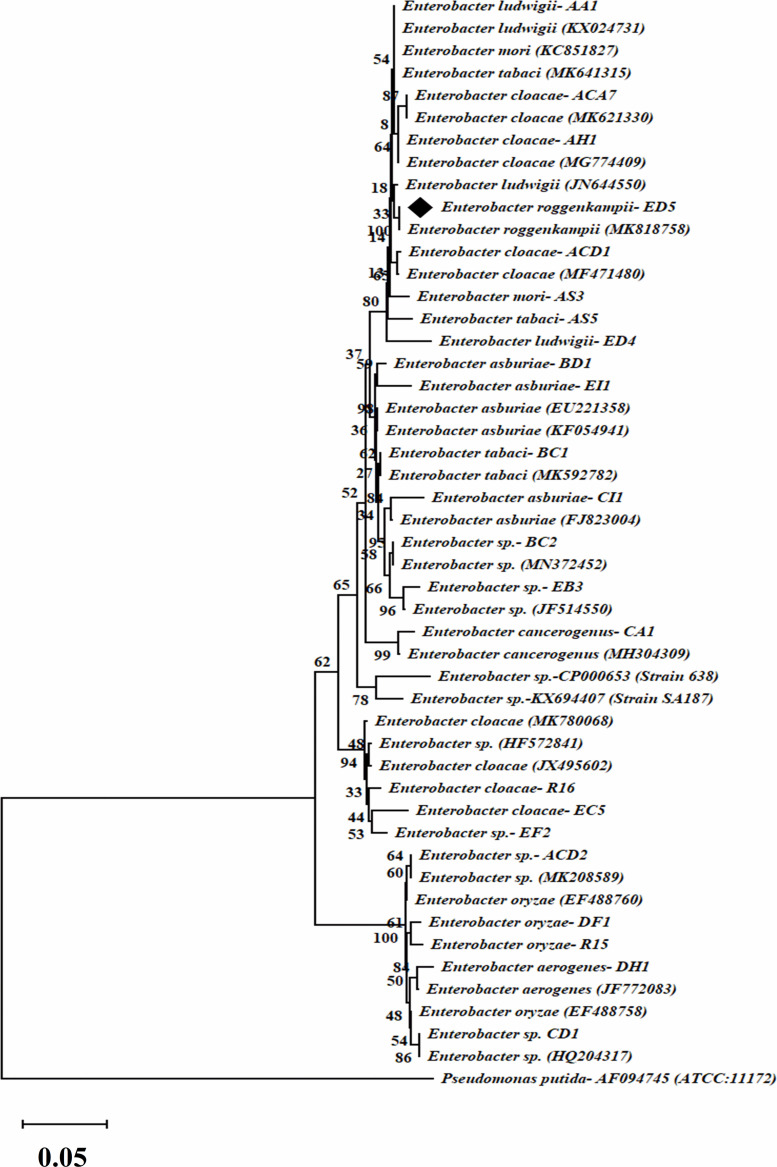
Dendrogram of 16S rRNA gene sequences of selected twenty-three endophytic *Enterobacter* isolates. The evolutionary distance was calculated by the UPGMA technique. Bootstrap analysis of 1,000 replications is specified as % confidence values for specific branching. Bar indicates % similarity and *P. putida*, as an outgroup.

The phylogenetic tree was formed by a comparison of 16S rRNA gene partial sequences of the selected 23 isolates with the reference strains sequences of the NCBI GenBank public database. The phylogenetic tree which was created by 1000 bootstrap sampling showed two major sets and *Pseudomonas putida* strain was employed as the reference strain to divide *Enterobacter* strains (Figure 2).

### *acdS* and *nifH* and Genes Amplification

**FIGURE 3:**
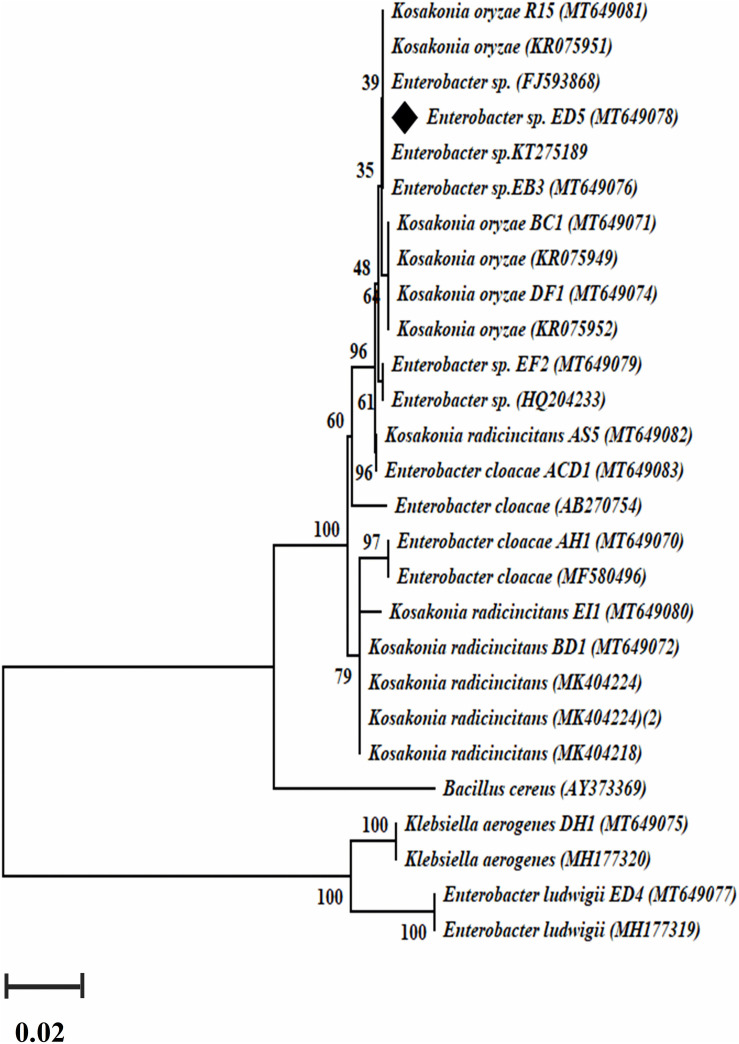
The dendrogram was created by a *nifH* gene sequences of the amplified *Enterobacter* strains by the neighbor-joining method.

Genomic DNA of all selected 23 endophytic strains was used to amplify *nifH and acdS* genes. Only 14 out of the 23 strains were confirmed positive *nifH* gene amplification, with a band size of 360 bp (Supplementary Figure S2) and a dendrogram was also created (Figure 3), whereas 12 confirmed positive *acdS* genes amplification with a band size of ∼ 755 bp (Supplementary Figure S3). All positive *nifH and acdS* strains were cloned and sequenced. After sequencing a BlastN search was finished and found all the sequenced clones were similar to the *nifH* gene sequences of NCBI GenBank database. In the case of *acdS* gene, only some sequenced clones showed similarity with *acdS* gene of the NCBI GenBank database and sequences not submitted. The identified *nifH* sequences were deposited in NCBI GenBank with accession numbers (MT649070-MT649083).

### Colonization Study of GFP-Tagged Endophytic ED5 Strain on Sugarcane

**FIGURE 4:**
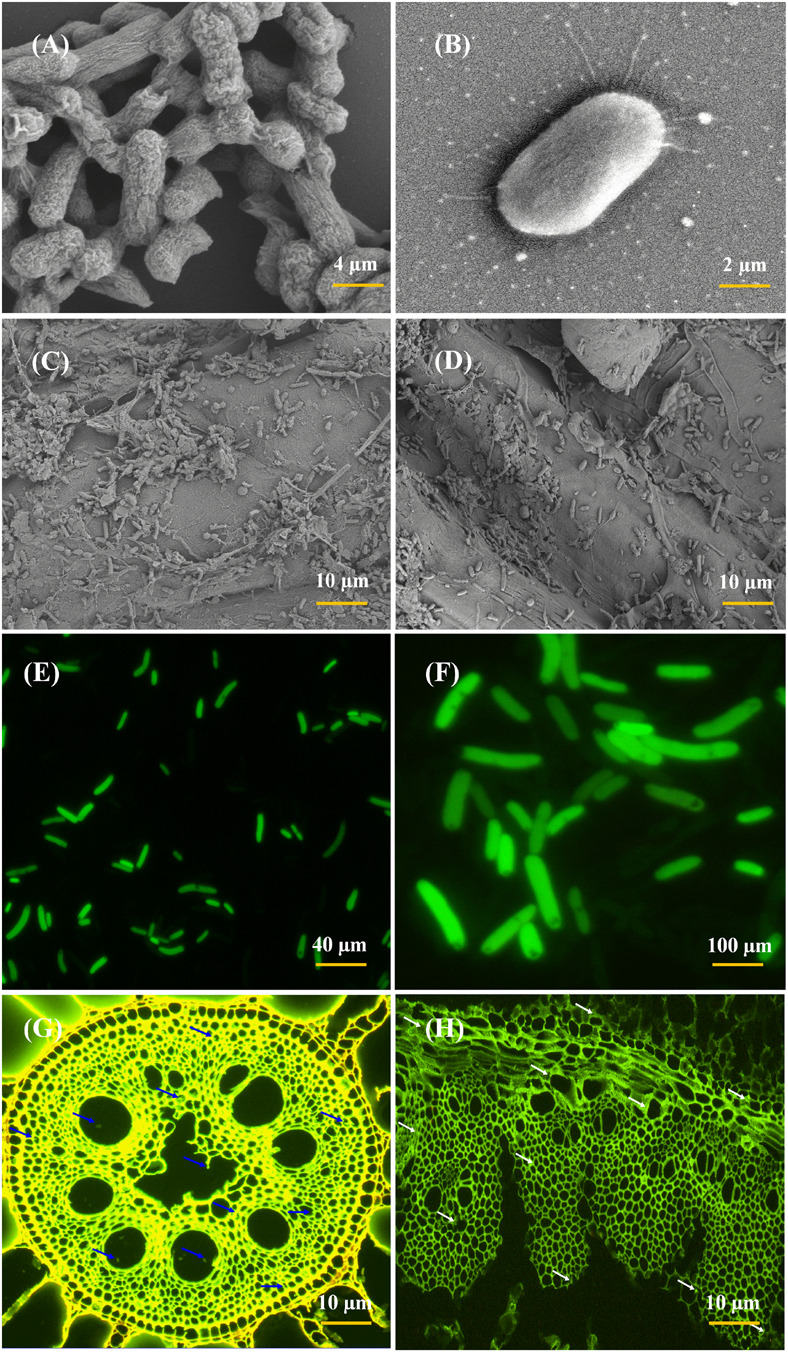
Scanning electron microscopy (SEM) and CLSM micrographs of most efficient endophytic *E. roggenkampii* ED5 strain and its colonization in sugarcane plant parts at the root and stem regions. Panels **(A,B)** is the SEM images showing the morphology of ED5 strain and, **(C,D)** is the colonization images obtained after the inoculation of ED5 strain in root and stem tissues of sugarcane. Panels **(E,F)** showing the CLSM micrographs of GFP-tagged endophytic ED5 strain, and **(G,H)**, showing the colonization in the roots and stems of sugarcane by GFP-tagged *E. roggenkampii* ED5. CLSM images showing the selected strain ED5 in green dots of auto-fluorescence in both root and stem tissues, respectively, and bacterial cells are specified by blue and white arrowheads. Both micrographs confirmed the colonization of inoculated endophytic *E. roggenkampii* ED5 strain in sugarcane.

The root colonization and colony morphology of ED5 strain was examined by SEM and CLSM (Figure 4), as this bacterium confirmed many PGP traits, excellent nitrogen-fixing potential, antifungal activity against plant pathogens, as well as survived in various abiotic stress circumstances. These techniques helped to study the interaction mechanism of the potential strains. In this study, *E. roggenkampii* ED5 strain was chosen for localization assessment in sugarcane cultivar with SEM and CLSM. Figures 4C,D, SEM results confirmed the colonization of *E. roggenkampii* in both stem and root tissues of sugarcane.

Whereas the GFP-tagged ED5 isolate transferred in sugarcane plants was also observed after 3 days of incubation and bacteria colonization was spotted as a green circle in all over of the plant stem and root tissues (Figures 4G,H). The density of ED5 strain had increased after incubation, and colonization of GFP-tagged strain was detected through the green auto-fluorescence produced as small dots in both roots and stems plant parts (Figure 4).

### Plant Growth Parameters

All physiological parameters (chlorophyll content, leaf area, plant height, root weight, shoot weight, photosynthesis, and transpiration rate) were significantly increased by inoculation of strain ED5 compared to control in GT11sugarcane cultivar at 30 and 60 days (Table 3).

**TABLE 3:** Evaluation of *E. roggenkampii* ED5 strain on the plant growth parameters of sugarcane under greenhouse conditions after inoculation at 30 and 60 days.

Parameters	Sugarcane cultivar GT11			
	Control	Treatment	Control	Treatment
	30 days		60 days	
Chlorophyll (SPAD units)	13.40 ± 0.20^*d*^	15.09 0.23^*d*^	28.60 ± 0.43^*c*^	32.04 ± 0.48^*cd*^
Leaf Area (cm^2^)	312.62 ± 4.69^*a*^	492.43 ± 7.39^*a*^	670.93 ± 10.07^*a*^	727.75 ± 10.92^*a*^
Height (cm)	21.07 ± 0.32^*c*^	22.58 ± 0.34^*c*^	25.09 ± 0.38^*c*^	24.08 ± 0.36^*d*^
Root Weight (g)	1.30 ± 0.02^*f*^	1.91 ± 0.03^*e*^	8.83 ± 0.13^*d*^	9.63 ± 0.14^*e*^
Shoot Weight (g)	2.41 ± 0.04^*ef*^	4.11 ± 0.06^*e*^	21.57 ± 0.32^*c*^	26.29 ± 0.39^*cd*^
Photosynthesis (μmol m^–2^ s^–1^)	5.48 ± 0.08^*e*^	26.48 ± 0.40^*c*^	9.23 ± 0.14^*d*^	33.41 ± 0.50^*c*^
Transpiration rate (mmol m^–2^ s^–1^)	1.07 ± 0.02^*f*^	1.25 ± 0.02^*e*^	1.17 ± 0.02^*e*^	2.45 ± 0.04^*e*^
gsw-stomatal conductance (mmol m^–2^ s^–1^)	27.47 ± 0.41^*b*^	43.96 ± 0.66^*b*^	39.30 ± 0.59^*b*^	75.21 ± 1.13^*b*^
SEM	1.18	1.86	2.53	2.75
CD (*P* = 0.05)	3.54	5.58	7.58	8.26
CV (%)	4.30	4.20	4.40	4.10

*Means with the same alphabets inside a column are not significantly different (p ≤ 0.05) according to DMRT (Duncan’s Multiple Range Test). SEM, standard error of the difference between means; CD, critical difference; CV, coefficient of variation*.

### Genomic Properties of ED5 Strain

**FIGURE 5:**
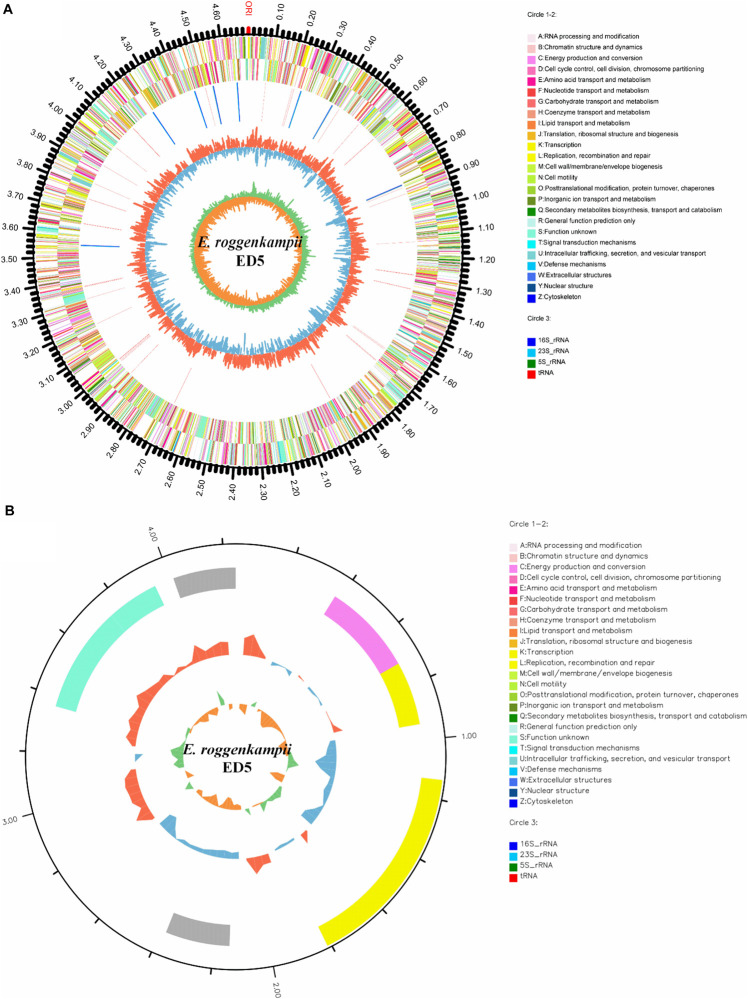
Circular representation of chromosome and plasmid of endophytic nitrogen-fixing *E. roggenkampii* ED5 strain isolated from sugarcane root. The inner and innermost rings display the GC content and skew. ORI; the origin of replication in chromosome map. **(A,B)**: A–Z, respectively, show the functional classification of the CDS genes in the chromosome and plasmid with the colors of the COG database and circle 3; different colors show different RNA types.

The general properties of the endophytic ED5 strain genome are presented in Table 4, which comprised 4,698,609 base pairs of a circular chromosome with an average 56.05% G + C content. There were about 4349 predicted CDSs (Figure 5A). In addition, the *E. roggenkampii* genome included 83 tRNA and 25 rRNA (9, 5S; 8, 16S, and 8, 23S) genes. The CDSs number allocated to the KEGG, COG, and GO database were 2839, 4028, and 2949 (Supplementary Figures S4–S6). And a circular plasmid with 4242 base pairs of DNA and the G + C content of 45.66% (Table 4). Plasmid genome annotations estimated protein-coding with 6 genes, and results involved mRNA-degrading endonuclease, hypothetical protein, a transcriptional regulator, and RNA polymerase (Figure 5B). Here, we used Island Path-DIMOB, PHAST, and Minced software to predict the presence of 7 gene islands, 5 CRISPR, and 2 prophages in the ED5 genome. Clustered regularly interspaced short palindromic repeats (CRISPR) are hypervariable loci extensively dispersed in bacteria which offer acquired resistance toward foreign genetic elements. It is composed of many short and conserved repeat regions and spacers. A total of 5 CRISPRs were predicted from the genome of strain ED5 with 25 bp shortest and 43 bp longest direct repeat sequences. Prophages are repeatedly confined in sequenced bacterial genomes through a simple semantic script and contain 90 CDS genes, mainly related to hypothetical protein, cold shock-like protein, phage tail protein, DNA polymerase V subunit UmuC, etc. Whereas, gene islands contain 160 CDS genes, mainly related to pyrimidine utilization protein, hypothetical protein, Type VI secretion protein, etc. (Table 4). A complete genome sequence of this strain has been submitted at Gen-Bank/EMBL/DDBJ with accession numbers CP058253–CP058254.

**TABLE 4:** Genome characteristic of endophytic strain *E. roggenkampii* ED5.

Characteristics	Value
Genome size (bp)	4,702,851
GC content (%)	56.05
Topology	Circular
Chromosome size (bp)	4,698,609
Plasmid size (bp)	4242
Plasmid GC content (%)	45.66
Chromosome	1
Plasmid	1
tRNA	83
rRNA (5S, 16S, 23S)	9, 8, 8
CDS (chromosome, plasmid)	4,343,6
Protein-coding genes (CDS)	4,349
Genomic islands	7
CRISPR	5
Prophage	2
Genes assigned to NR	4347
Genes assigned to Swiss-Prot	3818
Genes assigned to COG	4028
Genes assigned to KEGG	2839
Genes assigned to GO	2949
Genes assigned to Pfam	3964

### Genome-Based Phylogeny of ED5 Strain

**FIGURE 6:**
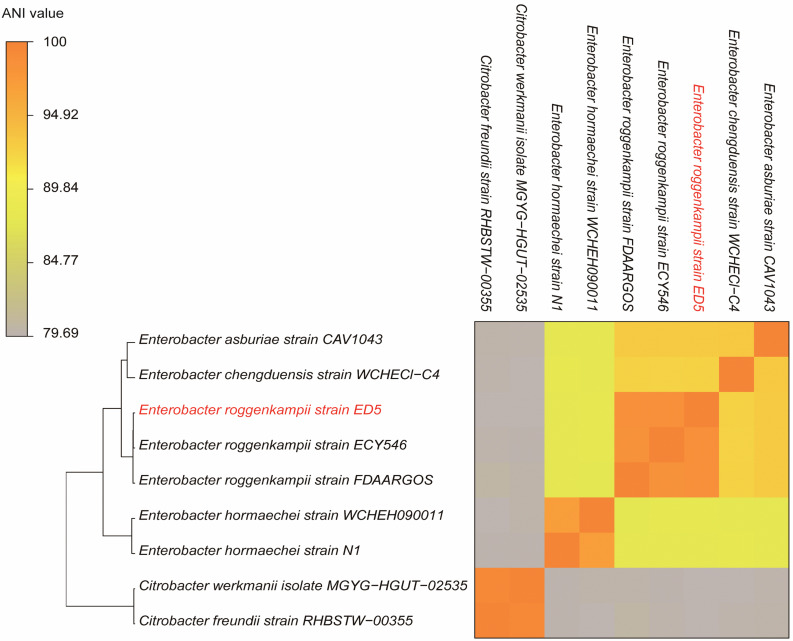
The heat maps of ANI (average nucleotide identity) between strain ED5 and phylogenetically eight closely related species. The ANI value among strain ED5 and *E. roggenkampii* FDAARGOS was 98.529% and *E. roggenkampii* ECY546 was 98.507%.

The ANI results showed that the genome of ED5 presents 98.5259% ANI to *E. roggenkampii* FDAARGOS and 98.507% ANI to *E. roggenkampii* ECY546, respectively. Cluster analysis showed that they were closely related. The ANI value of strain ED5 and other strains were less than 95%, the highest value was 93.1342% for *E. asburiae* CAV1043 and the lowest was 79.69% for *Citrobacter werkmanii* MGYG-HGUT-02535. These ANI results indicated that strain ED5 belongs to *E. roggenkampii* (Figure 6).

### Genes Efficiently Linked With PGP and Various Stress Tolerance in Endophytic ED5 Genome

**FIGURE 7:**
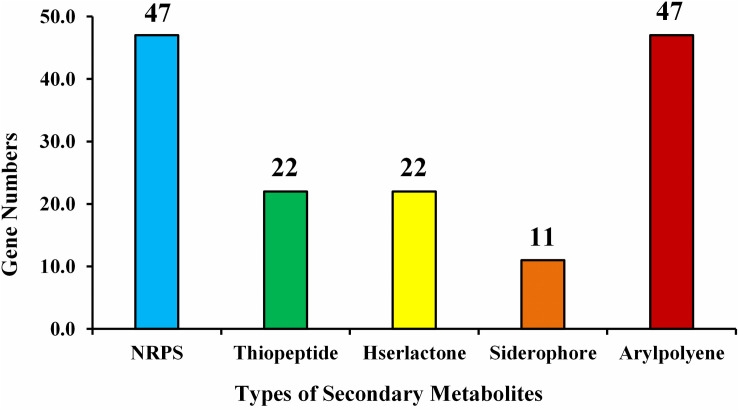
Type of secondary metabolites gene clusters in the genome of *E. roggenkampii* ED5 strain.

Examination of the recognized CDSs exposed the genome includes genes that encode proteins, related with nitrogen metabolism (*iscU*, *norRV*, and *gltBD*), ACC deaminase (*dcyD*), siderophores (*fes*, *entFS*, and *fepA*) plant hormones, phosphate metabolism, biofilm formation, root colonization, sulfur assimilation and metabolism, which are contributing in plant growth enhancement, were spotted (Table 5). The number of predicted gene clusters for secondary metabolite production such as NRPS, thiopeptide, Hserlactone, siderophore, and aryl-polyene are shown in Figure 7.

**TABLE 5:** Genes associated with PGP traits in *E. roggenkampii* ED5 genome.

PGP activities description	Gene name	Gene annotation	E.C. number	Chromosome location
Nitrogen metabolism				
Nitrogen fixation	*iscU*	Nitrogen fixation protein nifu and related proteins	–	3496630–3497016
Cyanate hydrolysis	–	Cyanate transport protein CynX	–	2160759–2161919
Nitrosative stress	*norR*	Anaerobic nitric oxide reductase transcription regulator NorR	–	3662018–3663532
	*norV*	Anaerobic nitric oxide reductase flavorubredoxin	–	3663720–3665162
Ammonia assimilation	*gltB*	Glutamate synthase (NADPH/NADH) large chain	1.4.1.13 1.4.1.14	4179778–4184238
	*gltD*	Glutamate synthase (NADPH/NADH) small chain	1.4.1.13 1.4.1.14	4184248–4185666
ACC deaminase	*dcyD*	1-Aminocyclopropane-1-carboxylate deaminase	4.4.1.15	2932253–2933239
**Siderophore**				
Siderophore enterobactin	*fes*	Enterochelin esterase and related enzymes		4687024–4688199
	*entF*	Enterobactin synthase subunit F	6.3.2.14	1205182–1209039
	*entS*	Enterobactin exporter entS	–	1212020–1213258
	*fepA*	Ferric enterobactin receptor	–	1201369–1203618
	–	Iron-enterobactin transporter ATP-binding protein	3.6.3.34	1209127–1209921
	–	Ferric enterobactin transport system permease protein FepD	–	1210907–1211911
	*entD*	Enterobactin synthase	6.3.2.14 2.7.8	1200642–1201283
**Plant hormones**				
IAA production	*trpCF*	Bifunctional indole-3-glycerol phosphate synthase/phosphoribosylanthranilate isomerase	4.1.1.48 5.3.1.24	2658686–2660044
	*trpS*	Tryptophanyl-trna synthetase	6.1.1.2	555353–556354
	*trpE*	Anthranilate synthase component	4.1.3.27	2655414–2657087
	*trpB*	Tryptophan synthase subunit beta	4.2.1.20	2660055–2661248
	–	Tryptophan synthase subunit alpha	–	2661248–2662057
	*trpGD*	Bifunctional glutamine amidotransferase/anthranilate phosphoribosyltransferase	4.1.3.27 2.4.2.18	2657087–2658682
Auxin biosynthesis	*mdcF*	Auxin Efflux Carrier	–	4290745–4291704
Phosphate metabolism	*pit*	Low-affinity inorganic phosphate transporter		4407682–4409181
	*pstS*	Phosphate ABC transporter substrate-binding protein	–	4682299–4683339
	*pstC*	Phosphate transporter permease subunit pstC	–	4683468–4684427
	*pstA*	Phosphate transporter permease subunit ptsA	–	4684427–4685317
	*pstB*	Phosphate ABC transporter ATP-binding protein	3.6.3.27	4685365–4686138
	*phoU*	Phosphate-specific transport system accessory protein phoU	–	4686165–4686890
	*ugpB*	Sn-glycerol-3-phosphate ABC transporter substrate-binding protein	–	299782–301074
	–	Glycerol-3-phosphate transporter permease	–	301181–302068
	*ugpE*	Glycerol-3-phosphate transporter membrane protein	–	302065–302910
	*phoA*	Alkaline phosphatase	3.1.3.1	978756–980171
	*phoE*	Phosphoporin protein E	–	912190–913242
	*phoB*	Two-component system, ompr family, phosphate regulon response regulator phoB	–	991972–992661
	*phoR*	Two-component system, ompr family, phosphate regulon sensor histidine kinase phoR	2.7.13.3	992683–993978
	*phoH*	Phosphate starvation-inducible protein phoh and related proteins	–	1276067–1277113
Biofilm formation	*tomB*	Biofilm formation regulator YbaJ	–	1073128–1073502
	*luxS*	*S*-ribosylhomocysteinase	4.4.1.21	3643176–3643691
	*efp*	Elongation factor P	–	412037–412603
	*flgN*	Flagella synthesis chaperone protein FlgN	–	1766271–1766666
	*flgM*	Flagellar biosynthesis protein FlgM	–	1766701–1766994
	*flgA*	Flagella basal body P-ring formation protein FlgA	–	1767088–1767747
	*flgB*	Flagellar biosynthesis protein FlgB	–	1767905–1768321
	*flgC*	Flagellar basal body rod protein FlgC	–	1768325–1768729
	*flgD*	Flagellar basal body rod modification protein	–	1768741–1769451
	*flgE*	Flagellar hook protein FlgE	–	1769478–1770686
	*flgF*	Flagellar basal body rod protein FlgF	–	1770707–1771462
	*flgG*	Flagellar basal body rod protein FlgG	–	1771474–1772256
	*flgH*	Flagellar basal body L-ring protein	–	1772305–1773012
	*flgI*	Flagellar basal body P-ring protein	–	1773025–1774122
	*flgJ*	Flagellar rod assembly protein/muramidase FlgJ	–	1774122–1775075
	*flgK*	Flagellar hook-associated protein FlgK	–	1775151–1776791
	*flgL*	Flagellar hook-associated protein FlgL	–	1776806–1777759
	*motB*	Flagellar motor protein MotB	–	2900550–2901479
	*motA*	Flagellar motor protein MotA	–	2901476–2902363
	*sacA*	Glycosyl hydrolase family 32	3.2.1.26	2381644–2383050
	*hfq*	RNA-binding protein Hfq	–	437637–437948
Sulfur assimilation	*cysZ*	Sulfate transporter CysZ	–	3374729–3375490
	*cysK*	Cysteine synthase A	2.5.1.47	1049190–1050236
	*cysM*	Cysteine synthase B	2.5.1.47	3380409–3381320
	*cysA*	Sulfate ABC transporter ATP-binding protein	3.6.3.25	3381439–3382533
	*cysW*	Sulfate/thiosulfate transporter permease subunit	–	3382523–3383398
	*cysU*	Sulfate/thiosulfate transporter subunit	–	3383398–3384231
	*cysP*	Sulfate transport system substrate-binding protein	–	3384231–3385244
	*cysC*	Adenylylsulfate kinase	2.7.1.25	3708229–3708834
	*cysN*	Sulfate adenylyltransferase	2.7.7.4	3708834–3710258
	*cysD*	Sulfate adenylyltransferase small subunit	2.7.7.4	3710268–3711176
	*cysH*	Phosphoadenosine phosphosulfate reductase	1.8.4.8 1.8.4.10	3713924–3714658
	*cysI*	Sulfite reductase subunit beta	1.8.1.2	3714674–3716386
	*cysJ*	Sulfite reductase subunit alpha	1.8.1.2	3716386–3718191
Sulfur metabolism	*cysC*	Adenylyl-sulfate kinase	2.7.1.25	3708229–3708834
	*cysN*	Sulfate adenylyltransferase	2.7.7.4	3708834–3710258
	*cysD*	Sulfate adenylyltransferase small subunit	2.7.7.4	3710268–3711176
	*cysG*	Siroheme synthase	2.1.1.107 1.3.1.76 4.99.1.4	3711186–3712544
	*cysH*	Phosphoadenosine phosphosulfate reductase	1.8.4.8 1.8.4.10	3713924–3714658
	*cysI*	Sulfite reductase subunit beta	1.8.1.2	3714674–3716386
	*cysJ*	Sulfite reductase subunit alpha	1.8.1.2	3716386–3718191
	*cysG*	Siroheme synthase	2.1.1.107 1.3.1.76 4.99.1.4	3711186–3712544
	*cysE*	Serine *O*-acetyltransferase	2.3.1.30	128779–129600
	*cysQ*	3′(2′),5′-bisphosphate nucleotidase CysQ	3.1.3.7	474494–475234
	*cysK*	Cysteine synthase	2.5.1.47	1049190–1050236
	*cysS*	Cysteine–tRNA ligase	6.1.1.16	1117790–1119175
	*cysZ*	Sulfate transporter CysZ		3374729–3375490
	*cysM*	Cysteine synthase B	2.5.1.47	3380409–3381320
	*cysA*	Sulfate ABC transporter ATP-binding protein	3.6.3.25	3381439–3382533
	*cysW*	Sulfate/thiosulfate transporter permease subunit	–	3382523–3383398
	*cysU*	Sulfate/thiosulfate transporter subunit	–	3383398–3384231
	*cysP*	Thiosulfate-binding protein	–	3384231–3385244
	*fdx*	Ferredoxin, 2Fe-2S type, ISC system	–	3493495–3493830
Antimicrobial peptide	*pagP*	Antimicrobial peptide resistance and lipid A acylation protein PagP	2.3.1.251	1249862–1250437
	*sapB*	Antimicrobial peptide ABC transporter permease SapB	–	2617964–2618929
Phenazine		Phenazine biosynthesis-like protein Phenazine biosynthesis-like protein		2296005–2296793 2296822–2297613
Hydrogen peroxide		Robbable hydrogen peroxide-inducible genes activator		3797368–3798234
**Synthesis of resistance inducers**				
2,3-butanediol	*alsD*	Alpha-acetolactate decarboxylase	4.1.1.5	1173252–1174034
	*ilvB*	Acetolactate synthase catalytic subunit	2.2.1.6	26918–28606
2,3-butanediol	*ilvH*	Acetolactate synthase isozyme 1 small subunit	2.2.1.6	28610–28897
2,3-butanediol	*ilvA*	Serine/threonine dehydratase	4.3.1.19	4097837–4098826
	*ilvC*	Ketol-acid reductoisomerase	1.1.1.86	4526147–4527622
	*ilvY*	Transcriptional regulator IlvY	–	4527775–4528665
	*ilvD*	Dihydroxy-acid dehydratase	4.2.1.9	4530258–4532108
	*ilvE*	Branched chain amino acid aminotransferase	2.6.1.42	4532169–4533098
	*ilvM*	Acetolactate synthase	2.2.1.6	4533117–4533380
Methanethiol	*metH*	Methionine synthase	2.1.1.13	251182–254865
Isoprene	*idi*	Isopentenyl-diphosphate Delta-isomerase	5.3.3.2	3832468–3833010
	*gcpE*	4-hydroxy-3-methylbut-2-en-1-yl diphosphate synthase	1.17.7.1 1.17.7.3	3469721–3470839
	*ispE*	4-(cytidine 5′-diphospho)-2-*C*-methyl-D-erythritol kinase	2.7.1.148	2728216–2729085
Hydrolase	–	Chitinase	3.2.1.14	1501603–1504335
	–	Chitinase II	3.2.1.14	1857320–1858573
	–	Chitinase	–	1505114–1506868
	–	Cellulase (glycosyl hydrolase family 5)	–	558158–559246
	*sacA*	Glycosyl hydrolase family 32	3.2.1.26	2381644–2383050
	*yxeP*	Hydrolase	3. –.–.–	2474994–2476115
	*ycjT*	Hypothetical glycosyl hydrolase	3.2.1. –	2602137–2604416
	*ribA*	GTP cyclohydrolase II	3.5.4.25	2641297–2641887
	*folE*	GTP cyclohydrolase I FolE	3.5.4.16	3154201–3154869
	*gdhA*	Glutamate dehydrogenase (NADP+)	1.4.1.4	1866296–1867639
	*bglA*	6-phospho-beta-glucosidase	3.2.1.86	38745–40121
	*bglF*	PTS beta-glucoside transporter subunit EIIBCA	2.7.1. –	1677933–1679801
	*bglX*	Beta-glucosidase	3.2.1.21	1927588–1929963
	*malZ*	Alpha-glycosidase	3.2.1.20	997326–999143
	*xynB*	xylan 1,4-beta-xylosidase	3.2.1.37	534506–536116
	*amyA*	Alpha-amylase	3.2.1.1	165374–167404
Oxidoreductase	*–*	Superoxide dismutase	1.15.1.1	1974715–1975296
	*gpx*	Glutathione peroxidase	1.11.1.9	2154552–2155034
	*osmC*	Peroxiredoxin	–	2398835–2399263
	*DOT5*	Peroxiredoxin	1.11.1.15	3413969–3414439
Symbiosis-related	*bacA*	Undecaprenyl-diphosphatase	3.6.1.27	4002393–4003214
	*gcvT*	Glycine cleavage system protein T	2.1.2.10	3849289–3850332
	*phnC*	Phosphonate ABC transporter ATP-binding protein	3.6.3.28	382433–383221
	*tatA*	Protein translocase tata	–	4470409–4470663
	*pyrC*	Dihydroorotase	3.5.2.3	501711–502844
	*pyrC*	Dihydroorotase	3.5.2.3	1759073–1760119
	*zur*	Transcriptional repressor	–	318300–318812
**Root colonization**				
Chemotaxis	*cheZ*	Protein phosphatase CheZ		2884011–2884655
	*cheY*	Two-component system response regulator	–	2884666–2885055
	*cheB*	Chemotaxis response regulator protein-glutamate methylesterase	3.1.1.61	2885073–2886122
	*cheR*	Chemotaxis protein-glutamate *O*-methyltransferase	2.1.1.80	2886119–2886985
	*cheW*	Chemotaxis protein CheW	–	2897986–2898489
	*cheA*	Chemotaxis protein CheA	2.7.13.3	2898509–2900539
	*cheV*	Chemotaxis protein CheV	–	3237160–3238164
	*tsr*	Methyl-accepting chemotaxis protein I	–	609842–611506
	*trg*	Chemotaxis protein	–	2243792–2245465
	*aer*	Chemotaxis protein	–	478104–2479639
	*tar*	Methyl-accepting chemotaxis protein II	–	2888651–2890318
	*mcp*	Methyl-accepting chemotaxis protein	–	3943578–3945125
Motility	*flhE*	Flagellar protein flhE	–	2880251–2880643
	*flhA*	Flagellar biosynthesis protein FlhA	–	2880643–2882721
	*flhB*	Flagellar biosynthesis protein FlhB	–	2882714–2883769
	*flhC*	Transcriptional activator FlhC	–	2902487–2903065
	*flhD*	Transcriptional regulator	–	2903068–2903418
	*fliY*	Cystine ABC transporter substrate-binding protein	–	2933346–2934146
	*fliZ*	Flagellar regulatory protein FliZ	–	2934233–2934784
	*fliA*	RNA polymerase sigma factor FliA	–	2934839–2935558
	*fliB*	Hypothetical protein	–	2935687–2936892
	*fliC*	Flagellin	–	2936955–2937779
	*fliD*	Flagellar filament capping protein FliD	–	2938181–2939605
	*fliS*	Flagellar protein FliS	–	2939627–2940031
	*fliT*	Flagellar biosynthesis protein FliT	–	2940037–2940411
	*fliE*	Flagellar hook-basal body complex protein FliE	–	2942532–2942846
	*fliF*	Flagellar M-ring protein FliF	–	2943123–2944754
	*fliG*	Flagellar motor switch protein FliG	–	2944747–2945745
	*fliH*	Flagellar assembly protein H	–	2945738–2946445
	*fliI*	Flagellum-specific ATP synthase FliI	3.6.3.14	2946445–2947815
	*fliJ*	Flagellar protein FliJ	–	947837–2948280
	*fliK*	Flagellar hook-length control protein	–	2948277–2949509
	*fliL*	Flagellar basal body-associated protein FliL	–	2949616–2950086
	*fliM*	Flagellar motor switch protein FliM	–	2950091–2951095
	*fliNY*	Flagellar motor switch protein FliN	–	2951092–2951505
	*fliOZ*	Flagellar biosynthesis protein FliO	–	2951508–2951882
	*fliP*	Flagellar biosynthetic protein FliP	–	2951882–2952619
	*fliQ*	Flagellar export apparatus protein FliQ	–	2952629–2952898
	*fliR*	Flagellar biosynthesis protein FliR	–	2952906–2953691
Adhesive structure	*pilT*	Type IV pili twitching motility protein PilT	–	3897208–3898050
	*pilD*	Prepilin peptidase	3.4.23.43 2.1.1.	1500548–1501345
	*hofC*	Type IV pilin biogenesis protein	–	771182–772183
Adhesin production	*pgaA*	Poly-beta-1,6 *N*-acetyl-D-glucosamine export porin PgaA	–	3922273–3924732
	*pgaB*	Outer membrane *N*-deacetylase	3.5.1. –	3924744–3926678
	*pgaC*	Poly-beta-1,6 *N*-acetyl-D-glucosamine synthase	2.4.1. –	3926671–3927999
	*pgaD*	Poly-beta-1,6-*N*-acetyl-D-glucosamine biosynthesis protein PgaD	–	3927999–3928430

Also, genes involved in plant resistance response, i.e., antimicrobial peptide, synthesis of resistance inducers, hydrolase genes such as chitinase, cellulase, α- amylase, GTP cyclohydrolase, glutamate dehydrogenase, xylan 1,4beta-xylosidase, and glucosidase, whereas, oxidoreductases genes such as superoxide dismutase (SOD), glutathione peroxidase (GPX) and peroxiredoxin (PRXS) were also categorized (Table 5). Strain ED5 genome predicted some key genes of volatile substances such as 2,3-butanediol (*alsD* and *ilvABCDEHMY*), methanethiol (*metH* and *idi*) and isoprene (*gcpE* and *ispE*) and might be involved in biocontrol mechanism of strain ED5 (Table 5). Some symbiosis-related genes were also observed in strain ED5 genome, which might play a role in the establishment of symbiosis with the sugarcane plant (Table 5).

**FIGURE 8:**
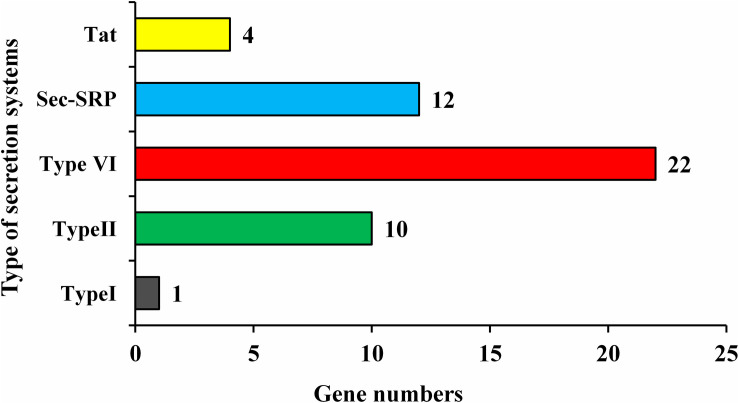
Types of secretion system present in strain ED5 genome.

*Enterobacter roggenkampii* genome study also confirmed the existence of numerous genes involved in different abiotic stresses tolerance, mainly, the cold shock (*cspA*), heat shock (*smpB hslR*, *ibpA*, *ibpB*, and *hspQ*), drought resistance (*nhaA*, *chaABC*, *proABPQSVWX*, *betABT*, *gabD*, *trkAH*, *kup*, and *kdpABCDEF*), and heavy metals (cobalt, zinc, cadmium, magnesium, copper, mercury, lead, and manganese) resistance were identified (Table 6). Pathogenic and non-pathogenic bacteria secrete some protein-like virulence factors to adapt and survive in their living host. In this study, strain ED5 genome showed five types of secretion systems such as Type I, Type II, Type VI, Sec-SRP, and twin-arginine translocase (Tat), involving 49 genes by using Diamond Version 0.8.35 software (Figure 8).

**TABLE 6:** Genes involved in different abiotic stresses in the *E. roggenkampii* ED5 genome.

Activity description	Gene name	Gene annotation	E.C. number	Chromosome location
Cold-shock protein	*cspA*	Cold-shock protein CspE	–	1250628–1250837
	*cspA*	Cold shock-like protein CspF	–	2524947–2525147
	*cspA*	Cold shock-like protein CspC	–	2829049–2829258
Heat shock proteins	*smpB*	SsrA-binding protein	–	3578158–3578640
	*hslR*	Heat-shock protein Hsp15	–	4329724–4330125
	*ibpA*	Heat-shock protein	–	10924–11379
	*ibpB*	Heat-shock protein IbpB	–	11516–11944
	*hspQ*	Heat-shock protein HspQ	–	1617738–1618055
**Heavy metal resistance**				
Cobalt-zinc-cadmium resistance	*czcD*	Cobalt-zinc-cadmium efflux system protein	–	1346027–1346959
Magnesium transport	*corA*	Magnesium transporter CorA	–	4489170–4490120
	*corC*	Magnesium and cobalt transporter	–	1274635–1275558
	*cobA*	Cob(I)yrinic acid a,c-diamide adenosyltransferase	2.5.1.17	2652149–2652739
Copper homeostasis	*CopC*	Copper resistance protein C	–	2849097–2849468
	*CopD*	Copper resistance protein D	–	2848226–2849095
	*cusA*	Cu(I)/Ag(I) efflux system membrane protein CusA/SilA	–	2326056–2329196
	*cusB*	Membrane fusion protein, Cu(I)/Ag(I) efflux system	–	2329207–2330454
	*cusF*	Cu(I)/Ag(I) efflux system periplasmic protein CusF	–	2330466–2330804
	*cusC*	Outer membrane protein, Cu(I)/Ag(I) efflux system	–	2330833–2332218
	*cusR*	Two-component system, OmpR family, copper resistance phosphate regulon response regulator CusR	–	2332380–2333063
	*cusS*	Two-component system, OmpR family, heavy metal sensor histidine kinase CusS	2.7.13.3	2333053–2334507
	*copA*	Cu^+^-exporting ATPase	3.6.3.54	1099416–1101914
Zinc homeostasis	*znuA*	Zinc ABC transporter substrate-binding protein	–	2864950–2865981
	*znuC*	Zinc ABC transporter ATP-binding protein ZnuC	3.6.3. –	2865972–2866727
	*znuB*	Zinc ABC transporter permease	–	2866724–2867509
	*–*	Zinc/manganese transport system substrate-binding protein	–	3653025–3653882
	*–*	Zinc/manganese transport system ATP-binding protein	–	3654781–3655434
	*zupT*	Zinc transporter ZupT	–	3987096–3987869
Zinc, cadmium, lead and mercury homeostasis	*zntA*	Zinc/cadmium/mercury/lead-transporting ATPase	3.6.3.3 3.6.3.5	282265–284436
Zinc homeostasis	*adhP*	Zinc-dependent alcohol dehydrogenase	1.1.1.1	2407431–2408441
	*htpX*	Zinc metalloprotease HtpX	3.4.24.	2834324–2835202
	*zntB*	Zinc transporter ZntB	–	2581221–2582204
Manganese homeostasis	*mntR*	Transcriptional regulator MntR	–	1415279–1415752
	*mntH*	Manganese transport protein	–	3360108–3361283
	–	Mn-containing catalase	–	2257406–2258278
Drought resistance	*nhaA*	Na^+^:H+ antiporter, NhaA family	–	683880–685010
	*chaC*	Cation transport protein ChaC	–	2718487–2719182
	*chaB*	Cation transport regulator	–	2719360–2719590
	*chaA*	Ca^2+^:H+ antiporter	–	2719861–2720961
	*proB*	Glutamate 5-kinase	2.7.2.11	913547–914650
	*proA*	Glutamate-5-semialdehyde dehydrogenase	1.2.1.41	914662–915915
	*proQ*	ProP effector	–	2837467–2838153
	*proV*	Glycine betaine/L-proline ABC transporter ATP-binding protein	3.6.3.32	3632168–3633370
	*proW*	Proline/betaine ABC transporter permease ProW		3633363–3634427
	*proX*	Glycine betaine ABC transporter substrate-binding protein		3634437–3635432
	*proP*	Proline/betaine transporter		389805–391307
	*proS*	Proline–tRNA ligase	6.1.1.15	865261–866979
	*betA*	Choline dehydrogenase	1.1.99.1	1187889–1189553
	*betB*	Betaine-aldehyde dehydrogenase	1.2.1.8	1189567–1191039
	*betT*	Choline/glycine/proline betaine transport protein	–	1191769–1193802
	*gabD*	Succinate-semialdehyde dehydrogenase [NADP (+)]	1.2.1.16 1.2.1.79 1.2.1.20	589996–591366
	*trkA*	Trk system potassium transport protein TrkA	–	4256588–4257964
	*trkH*	Trk system potassium uptake protein	–	4458220–4459671
	*trkH*	Trk system potassium uptake protein		3960701–3962176
	*kup*	Potassium transporter Kup	–	4660743–4662611
	*kdpE*	DNA-binding response regulator	2.7.13.3	1302671–1303348
	*kdpD*	Two-component sensor histidine kinase	3.6.3.12	1303345–1306032
	*kdpC*	Potassium-transporting ATPase subunit C	3.6.3.12	1306033–1306608
	*kdpB*	K^+^-transporting ATPase subunit B	3.6.3.14	1306621–1308669
	*kdpA*	Potassium-transporting ATPase subunit KdpA	–	1308688–1310367
	*kdpF*	K^+^-transporting ATPase subunit F	–	1310367–1310570

## Discussion

In China, farmer’s applying higher doses of chemical fertilizers especially N-fertilizers to enhance growth and yields of sugarcane, but the use of the higher amount of chemical fertilizers increases the production cost as well as have unfavorable results on the environment, causes severe soil and water pollution, the decline in beneficial microbial flora associated with PGP, and nitrogen mineralization, etc. (Herridge et al., 2008; Li and Yang, 2015; Singh et al., 2020). The main objective of this research work is lookup an endophytic microbe that fixes nitrogen for prolong periods in sugarcane as well as another crop. Therefore, here we have focused to isolate and identify only on root endophytic strains of *Enterobacter* genus, as this is an important genus of nitrogen fixation. A total of 23 endophytic *Enterobacter* strains were designated and identified with 16S rRNA gene sequencing with *E. roggenkampii* was the most prominent strain. Endophytic bacterial strains interact with the plant extra efficiently than rhizospheric bacteria and increasingly provide several benefits to the host plant, generally growth promotion, and tolerance to biotic and abiotic stresses, also carry the genes essential for BNF, to change dinitrogen gas (N_2_) into usable forms of nitrogen, ACC deaminase activity, P- solubilization, and produce plant hormones, for example, IAA (Gaiero et al., 2013; Beltran-Garcia et al., 2014; Lebeis, 2014; Santoyo et al., 2016; Maksimov et al., 2018; White et al., 2018).

The *Enterobacter* strains are well-known nitrogen fixers, plant colonizers, and highly resistant to biotic and abiotic stresses (English et al., 2010; Zhu et al., 2013; Sarkar et al., 2018; Macedo-Raygoza et al., 2019). All selected strains showed significant biocontrol activities against several pathogens used by dual culture method, and *E. roggenkampii* (ED5) showed highest antagonistic activity against *F. moniliforme*, *F. cubense*, *B. cinerea*, *C. paradoxa*, and *S. scitamineum* which indicates the potential application for management of diseases caused by various pathogens. Whereas, *E. roggenkampii* strain is unknown for the ability to produce secondary metabolites, various PGP traits including colonization ability, and environmental stresses. Greenhouse experiment confirmed that selected strain ED5 improve the growth of physical parameters in sugarcane. Because previously no information was reported to compare this strain, we need to go for complete genome sequencing and annotation of this endophytic strain, which offers a useful platform to study all nitrogen-fixing, PGP, and stress tolerance mechanisms. Here, in this study, a complete genomic analysis of ED5 strain identified several genes clusters related to antimicrobial peptide, synthesis of resistance inducers, and hydrolases, including *pagP*, *sapB*, *alsD*, *ilvABCDEHMY*, *metH*, *idi*, *gcpE*, *ispE*, *sacA*, *yxeP*, *ycjT*, *ribA*, *folE*, *gdhA*, *bglAFX*, *malZ*, *xynB*, *amyA*, and some unknown gene name. Identification of the genes associated with the production of antimicrobial compounds especially to stimulate the antibiotic production recommends the biocontrol ability of strain ED5 as well as its function as a different PGP trait and nitrogenase activity genes that can indirectly stimulate plant health by defeating the pathogens (Shariati et al., 2017). We identified several genes that are known to support the production of antimicrobial compounds and they additionally contained genes for chitinase, cellulase, and beta-glucosidase enzyme that damage the pathogenic fungi cell walls, and similar genes are also reported earlier in other strains (Cho et al., 2015; Shariati et al., 2017; Luo et al., 2018; Yang et al., 2019) as well as growth-stimulating volatile compounds, are produced by some of the most efficient PGPR strains, including *Enterobacter* spp. (Weilharter et al., 2011).

The endophytic PGP *Enterobacter* strains were used as microbial inoculants in many crops globally, to decrease the application of chemical fertilizers and increase the yield of the crops, in addition to maintaining soil fertility (Singh et al., 2017; Daur et al., 2018; de Zélicourt et al., 2018). Therefore, this study explored almost all PGP traits like nitrogen fixation, IAA, siderophore, phosphate, ACC, HCN, and ammonia production of the selected strains isolated from sugarcane root. Several other studies also showed that all PGP traits comprising bacterial strains from sugarcane used as bio-inoculants and increased sugarcane yield (Li et al., 2017; Singh et al., 2020). The genome of *E. roggenkampii* covers several genes contributing to plant-beneficial roles, such as ACC, siderophore, ammonia, IAA production, phosphate metabolism, and nitrogen fixation. The presence of important genes encoding for PGP mechanisms was also determined previously, and some related genes were informed by Asaf et al. (2018) and Kang et al. (2020).

Nitrogen is one of the essential micronutrients for plant growth, while nitrogen metabolism is the main metabolic activity of bacterial cells. Earlier, several Enterobacteriaceae for example *E. oryzae* Ola51^*T*^, *Enterobacter agglomerans*, and *E. cloacae* were accounted as nitrogen-fixers (Kreutzer et al., 1991; Peng et al., 2009; Laili et al., 2017). The *nifH* is a well-recognized functional gene and its amplification via degenerate primers is a convenient method to confirm the nitrogen-fixation capability of the strains (Zehr and Capone, 1996; Rosado et al., 1998). In this study, all endophytic bacteria established nitrogen-fixing potential through the ARA method in an N-free medium. However, only 14 strains confirmed *nifH* gene amplification at around 360 bp of band size. Most prominent strain *E. roggenkampii* genome encloses six nitrogen metabolism associated genes, i.e., *iscU*, *norRV*, and *gltBD* with one unknown gene name, which proved that the strain is directly connected with nitrogen metabolisms such as nitrogen fixation, cyanate hydrolysis, nitrosative stress, and ammonia assimilation. Gene *iscU* is responsible for nitrogen fixation protein *nifU* and related proteins; *nifU* protein contributes a major role in the Fe-S cluster congregation, which is necessary for nitrogen fixation (Smith et al., 2005). In contrast, Andrés-Barrao et al. (2017) reported *Enterobacter* sp. SA187 genome includes dissimilatory nitrate reduction genes apart from genes coding for the nitrogenase enzyme (*nifDHK*). *Klebsiella variicola* GN02 and *K. variicola* DX120E genome hold numerous genes associated with nitrogen fixation, for example, *nif* gene cluster (*nifHDK* and *nifLA*), nitrogen metabolism-regulatory genes (*ntrBC* and *glnD*), and ammonium carrier gene (*amtB*) (Lin et al., 2012, 2015; Biaosheng et al., 2019).

Phosphorus is another vital and limiting macronutrient for the plant’s production, along with nitrogen. Specific bacteria play an important part in supplying accessible inorganic phosphorous in the form of orthophosphate (PO_4_^3–^) to the plant, owing to phosphate is generally existing in the soil in the form of insoluble compounds and plants are only proficient to receive free orthophosphate (PO_4_^3–^) (Bergkemper et al., 2016). In the present study, 16 *Enterobacter* strains showed phosphate solubilization traits. Similar to our results, other *Enterobacter* strains such as *E. asburiae* (Gyaneshwar et al., 1999), *Enterobacter* sp. EnB1 (Delgado et al., 2014), *E. cloacae* SBP-8 (Singh et al., 2017), and *Enterobacter* sp. SA187 (Andrés-Barrao et al., 2017) have been also reported as phosphate solubilizers. The genome of ED5 includes 14 genes (*pit*, *pstABCS*, *phoUAEBRH*, and *ugpBE*, with one unknown gene name) coding for phosphate metabolism. The *Pit* system is constitutive, whereas *Pst* transporter is inhibited by phosphate and induced under phosphate limitation (Jansson, 1988). Andrés-Barrao et al. (2017) reported that the *Enterobacter* sp. SA187 genome comprises genes coding for phosphate uptake, low-affinity inorganic phosphate transporter, and phosphate starvation response.

Several helpful bacteria comprise PGP activity that is occurred by various mechanisms, such as inactivation or production of ACCD enzyme activity. PGPB including ACCD decreased the ethylene content in plants and encouraged root elongation (Penrose and Glick, 2003). In this study, all strains showed ACCD enzyme activity whereas, only 12 strains confirmed *acdS* gene amplification of ∼750–755 bp. Interestingly, one *dcyD* gene, coding for ACC deaminase, was present in *E. roggenkampii* genome. ACCD activity has been reported in many *Pseudomonas*, *Bacillus*, and *Mesorhizobium* strains, along with members of *Enterobacter* genus such as *E. cloacae* UW4, *E. cloacae* CAL2, *E. cancerogenus*, and *Enterobacter* sp. EN-21 (Shah et al., 1998; Holguin and Glick, 2001; Glick, 2014; Li et al., 2017; Kruasuwan and Thamchaipenet, 2018; Singh et al., 2020). IAA production from tryptophan by indole pyruvate is another approach of PGPB to improve plant growth (Taghavi et al., 2009). We observed that all endophytic strains were capable to synthesize IAA, and *E. roggenkampii* holds *trpBCEFS*, and *trpGD* genes code for enzymes concerned in this pathway. Moreover, we identified one gene auxin efflux carrier (*mdcF*) related to auxin biosynthesis, confirm their potential to be used as growth regulators. In similar to our findings, previously also well- recognized that the existence of tryptophan associated genes in genomes of bacteria is related to IAA production (Tadra-Sfeir et al., 2011; Gupta et al., 2014). As reported in *Enterobacter* strain 638 (Taghavi et al., 2010) and *E. cloacae* UW5 (Coulson and Patten, 2015) improved IAA levels and stimulate root development. Asaf et al. (2018) found tryptophan biosynthesis genes (*trpABD*) involved in IAA production was found in *Sphingomonas* sp. LK11 genome (Asaf et al., 2018).

PGPB developed a particular method for iron absorption by siderophores production, which transfers this component into their cells (Arora et al., 2013). In this study, 13 isolates showed positive siderophore production. Siderophore production by these strains expects importance for iron nutrition of plants matures in iron-limited situations. ED5 strain demonstrated strong siderophore activity and siderophore enterobactin (*fes*, *entFS*, and *fepA*) biosynthesis pathway was also observed in its genome study. Consistent with this study, the siderophore enterobactin pathway (*fepEGDC*) was detected in *E. cloacae* SBP-8 (Singh et al., 2017) and *Bacillus subtilis* EA-CB0575 genomes (Franco-Sierra et al., 2020).

A biofilm is a surface-linked efficient microorganism confined by a polymeric matrix including self-making exopolysaccharides, extracellular DNA and proteins related through the biotic surface (Hall-Stoodley and Stoodley, 2002; Vlamakis et al., 2013; Teschler et al., 2015). A large amount of beneficial microbial community’s structure recommended a biofilm, and some of the most fascinating recommended helpful biofilms are used in agriculture (Kumar et al., 2019; Singh M. P. et al., 2019). In the endophytic and rhizospheric zone, several kinds of bacterial species involve plant roots and create a biofilm which offers benefits to each other. Nowadays, plant-associated microorganisms have concerned a lot of interest because of the considerable effects of plant health and productivity (Bogino et al., 2013). Some of the PGPR showed antagonistic activity in response to phytopathogens by starting biofilm-like assemblies that have been previously reported to *Bacillus cereus* (Xu et al., 2014), *Paenibacillus* (Timmusk et al., 2015), and *Pseudomonas stutzeri* (Wang et al., 2017). In the biofilm, a cell-to-cell communication raises the gene expression of both up-down regulation, for enhancing the adaptation of microorganisms in both biotic and abiotic environments. In the *E. roggenkampii* genome, 21 genes, *tomb*, *luxS*, *efp*, *flgABCDEFGHIJKLNM*, *motAB*, *sacA*, and *hfq* which are associated to exopolysaccharides biosynthesis, protein, and biofilm formation were also found (Gupta et al., 2014; Ju et al., 2018).

Beneficial microorganism generally colonizes on the surface and inside tissues of various sections in the plant, i.e., root, stem, and leaf, the place they stay either commensally or perform helpful features (Johnston and Raizada, 2011). The interactions between beneficial microbes of the host plant might play an essential part in the achievement of microbial bioinoculant for improving the production of crops, but there is no strong thought on the entire role of colonization on the plant microbiome. The GFP pictures showed a signal of the occurrence of *E. roggenkampii* in the intercellular regions also as cell aggregates or isolated single cells in the root of sugarcane (Li et al., 2017; Singh et al., 2020). The SEM images confirmed of selected inoculated strain *E. roggenkampii* in stem and roots specified that the forms of adherence in sugarcane, and similar observation were also found in other endophytic strains like *Paenibacillus polymyxa*, *Rhizobium* sp. and *Burkholderia* sp. (Timmusk et al., 2005; Singh et al., 2009). In this study, we also clearly confirmed the result of root colonization genes present in *E. roggenkampii* through genome analysis. We recognized a great number of root colonization genes present in *E. roggenkampii* at different stages: chemotaxis (*cheABRVWYZ*, *tsr, trg*, *aer*, *tar*, and *mcp*) for signal transduction, motility (*flhEABCD*, *fliABCDEFGHIJKLMNOPQRSTYZ*) for genes regulation, adhesive structure (*pilDT*, and *hofC*), play a significant function in host–microbes interactions (Dorr et al., 1998; Krause et al., 2006) and adhesin production (*pgaABCD*). A similar observation was also reported by Cho et al. (2015).

Hydrogen sulfide (H_2_S) production by PGPR has been described to improve plant growth, and root colonization (Dooley et al., 2013). H_2_S, as an important molecule with beneficial effects on P-solubilization, responds with ferric phosphate to crop ferrous with the release of phosphate (Shariati et al., 2017). Sulfur is a vital nutrient for plant increase and development, along with related to stress tolerance in plants (Gill and Tuteja, 2011), because lacking sulfur in plant cause severe losses in crop yield and production. In the genome of *E. roggenkampii* ED5 genes associated with sulfate transporters (*cysACDJHIKMNPUWZ*) were found. Earlier, the *cysP* gene function was verified with a strain *Escherichia coli* transformed by a plasmid expressing *B. subtilis cysP* gene through a mutated sulfate transport (Mansilla and de Mendoza, 2000). The operon determined by *cysP* gene in *B. subtilis* is accountable for sulfur metabolism, for example, the sulfate adenylyltransferase gene (Aguilar-Barajas et al., 2011). The strain *E. roggenkampii* genome encodes the set of genes that are responsible for H_2_S biosynthesis, including the *cysACDEGHIJKMNPQSUWZ*, and *fdx* genes presented in assimilated sulfate reduction. The existence of an ATP-binding transporter gene that contains periplasmic binding proteins *cysP*, *cysW*, and *cysA* were determined in the genome of ED5 that discovered these genes might be elaborated in the transportation of thiosulfate or inorganic sulfate to cells, earlier reported in *Pseudomonas* sp. UW4 (Duan et al., 2013). The occurrence of these genes in microorganisms has been associated with the oxidation of sulfur and sulfur-conjugated secondary metabolites (Kwak et al., 2014). Also, sulfur oxidation effects soil pH and successively recovers solubility of micronutrients, i.e., N, P, K, Mg, and Zn (Vidyalakshmi et al., 2009). Hence, this type of beneficial endophytic microbes can offer enhanced mineral achievement and distribution to the host plants (Kang et al., 2020).

Abiotic stresses are highly injurious for the plants, the most unfavorable influence from physiological to the molecular level of the plants. Drought and heavy metal stress greatly decrease crop yield (Khan et al., 2018; Kang et al., 2020). Drought is the leading reason for preventive crop production over massive areas of the Earth (Saleem et al., 2018), and estimated to cause severe plant growth difficulties for above 50% of the arable lands in 2050 (Vinocur and Altman, 2005; Kasim et al., 2013). However, heavy metal recognized as a principal hazard in many terrestrial ecosystems globally (Shahid et al., 2015). Currently, significant industrialization will directly increase the heavy metals in soils, that have damaging outcomes on plant growth and productiveness (Forstner and Wittmann, 2012). The numerous micro-organisms have an intrinsic ability to manage abiotic stresses and improve plant growth. Earlier, several PGPR genera have been described to succeed drought and heavy metal stresses through various mechanisms, and to improve plant tolerance especially abiotic stresses (Dary et al., 2010; Hassan et al., 2017), as well as to improve the production of crops (Sessitsch et al., 2013).

A number of mechanisms have been described earlier about the heavy metal stress resistance of many microbial species (Kunito et al., 1996). Investigation of the ED5 genome discovered the occurrence of various genes presented in the homeostasis of heavy metals like copper, zinc, manganese, cadmium, etc. A genes *czcD*, *znuABC, zupT*, *zntAB* are found and these proteins were linked to the role of generating tolerance to about divalent cations with cobalt, zinc, and cadmium, owing to its capability to automatically produce an efflux of metal ions (Mima et al., 2009). Additionally, the magnesium and cobalt transport genes *corAC* and *cobA* were found in the ED5 genome. These genes have been stated to be elaborate in manganese transport, *mntR and mntH* genes considered the primary manganese transporters in bacteria (Kang et al., 2020). The copper (Cu) is an essential component for biological progressions, and a similar metal cofactor of several enzymes like monooxygenases, dioxygenases, and SOD (Giner-Lamia et al., 2012). The genes found in ED5 genome like *copCD*, *cusABCFRS*, and *copA*, have similar operon has been existing in bacterial species of *Pseudomonas psychrotolerans*, *Pseudomonas syringae*, *Xanthomonas campestris*, and *E. coli* (Duncan et al., 1985; Lee et al., 1992; Silver et al., 1993; Voloudakis et al., 1993; Cooksey, 1994; Kang et al., 2020). To further verify the ability of drought tolerance, we mentioned extraordinary resistance mechanisms in ED5, inclusive of tolerance to heavy metal, pH, and temperature stresses. Genes related to drought stress were determined in the ED5 genome are *nhaA*, *chaABC*, *proABPQSVWX*, *betABT*, *gabD*, *trkAH*, *kup*, *kdpABCDEF.*

## Conclusion

The present study selected the numbers of nitrogen-fixing endophytic strains of *Enterobacter* genus from the sugarcane roots. All strains exhibited many PGP traits, biocontrol activity, as well as tolerance to various environmental conditions, and *E. roggenkampii* ED5 was the most prominent strain among all. Therefore, the employ of efficient endophytic bacteria is an opportunity to improve crop yield and comprehensive genome sequencing of ED5 strain has revealed many prospects to study this potential endophytic strain in the future. Also, the ED5 genome carried a set of universal genes that contributed to PGP, nitrogen fixation, and response to several stresses. So, it can be summarized that ED5 strain may be used as a possible alternate for chemical fertilizers and play an important part in improving ecosystem quality. However, field trials are required to explain the usability of the *E. roggenkampii* ED5 in the field earlier than it can be established as a plant growth promoter for utilizing in sustainable agriculture.

## Data Availability Statement

The datasets generated in this study can be found in online repositories. The names of the repository/repositories and accession number(s) can be found in the article/Supplementary Material.

## Author Contributions

D-JG, RS, PS, and Y-RL planned the proposal and experiments. RS, PS, and D-JG accomplished the experiments. AS, D-PL, and X-PS performed the data examination. L-TY, Y-XX, and Y-RL contributed to study and resources. D-JG, RS, and PS wrote the original manuscript. Y-XX and Y-RL reviewed and edited the manuscript. All authors contributed to the article and approved the submitted version.

## Conflict of Interest

The authors declare that the research was conducted in the absence of any commercial or financial relationships that could be construed as a potential conflict of interest.

## Footnotes

1 www.majorbio.com

2 https://blast.ncbi.nlm.nih.gov/Blast.cgi
