# Supplementary material for: Complete Genome Sequence of Enterobacter roggenkampii ED5, a Nitrogen Fixing Plant Growth Promoting Endophytic Bacterium With Biocontrol and Stress Tolerance Properties, Isolated From Sugarcane Root
Source: Front Microbiol. 2020 Sep 22;11:580081. doi: 10.3389/fmicb.2020.580081 (PMC7536287; doi:10.3389/fmicb.2020.580081)
Supplement: Supplementary file 1 [file Data_Sheet_1.docx]

## Supplementary Tables

**TABLE S1** List of media used for isolation of endophytic *Enterobacter* strains.

1. **Ashby’s Glucose Agar (gm L^-1^)**

| Glucose | 20 |
| --- | --- |
| Dipotassium phosphate | 0.2 |
| Magnesium sulphate | 0.2 |
| Sodium chloride | 0.2 |
| Calcium carbonate | 5 |
| Potassium sulphate | 0.1 |
| Agar | 15 |
| Final pH | 7.4 |

1. **Ashby’s Mannitol Agar (gm L^-1^)**

| Mannitol | 20 |
| --- | --- |
| Dipotassium hydrogen phosphate | 0.2 |
| Magnesium sulphate | 0.2 |
| Sodium chloride | 0.2 |
| Calcium carbonate | 5 |
| Potassium sulphate | 0.1 |
| Agar | 15 |
| Final pH | 7.4 |

1. **Burk’s Medium (gm L^-1^)**

| Magnesium sulphate | 0.2 |
| --- | --- |
| Dipotassium hydrogen phosphate | 0.8 |
| Potassium dihydrogen phosphate | 0.2 |
| Calcium sulphate | 0.13 |
| Iron (III) Chloride | 1.45mg |
| Sodium molybdate | 0.253mg |
| sucrose | 20 |

1. **Jensen’s Broth, Granulated (gm L^-1^)**

| Magnesium sulphate | 0.5 |
| --- | --- |
| Dipotassium hydrogen phosphate | 1 |
| Sodium Chloride | 0.5 |
| Ferrous sulphate | 0.1 |
| Sodium molybdate | 0.005 |
| Calcium carbonate | 2 |
| sucrose | 20 |

1. **Yeast Mannitol Agar w/1.5% Agar (gm L^-1^)**

| Yeast extract | 1 |
| --- | --- |
| Mannitol | 10 |
| Sodium chloride | 0.1 |
| Calcium carbonate | 1 |
| Dipotassium hydrogen phosphate | 0.5 |
| Magnesium sulphate | 0.2 |
| Agar | 15 |
| Final pH | 6.8 |

1. **Nutrient Agar (gm L^-1^)**

| Trypsin | 10 |
| --- | --- |
| Beef extract | 3 |
| Sodium chloride | 5 |
| Agar | 15 |
| pH | 7.2 |

**TABLE S2** Primers sequence used for 16S rRNA, *nifH* and *acdS* genes amplification.

| **Gene** | **Primer** | **Sequence (5**′ **-------→ 3′)** | **Product size (bp**) | **PCR conditions** |
| --- | --- | --- | --- | --- |
| 16S | pA-F  pH-R | AGAGTTTGATCCTGGCTCAG  AAGGAGGTGATCCAGCCGCA | 1300 to 1600 | Initial temperature (95°C for 5m), start cycles (30), Denaturation (95°C for 1m), Annealing (55°C for 1m), Elongation (72°C for 1m), Final extensions (72°C for 5m). |
| *NifH* | PolF  PolR | TGCGAYCC-SAARGCBGACTC  ATSGCCATCATYTCRCCGGA | 360 | Initial temperature (94°C for 3m), start cycles (30), Denaturation (94°C for 1m), Annealing (55°C for 1m), Elongation (72°C for 1m), Final extensions (72°C for 10m). |
| *acdS* | ACD-F  ACD-R | GCAACAAGACGCGCAAGYTNGARTAYN T  GTGCATCGACTTGCCCTCRWANACNGG RT | 755 | Initial temperature (94°C for 4m), start cycles (35), Denaturation (94°C for 45s), Annealing (53°C for 45s), Elongation (72°C for 1m), Final extensions (72°C for 10m). |

## Supplementary Figures


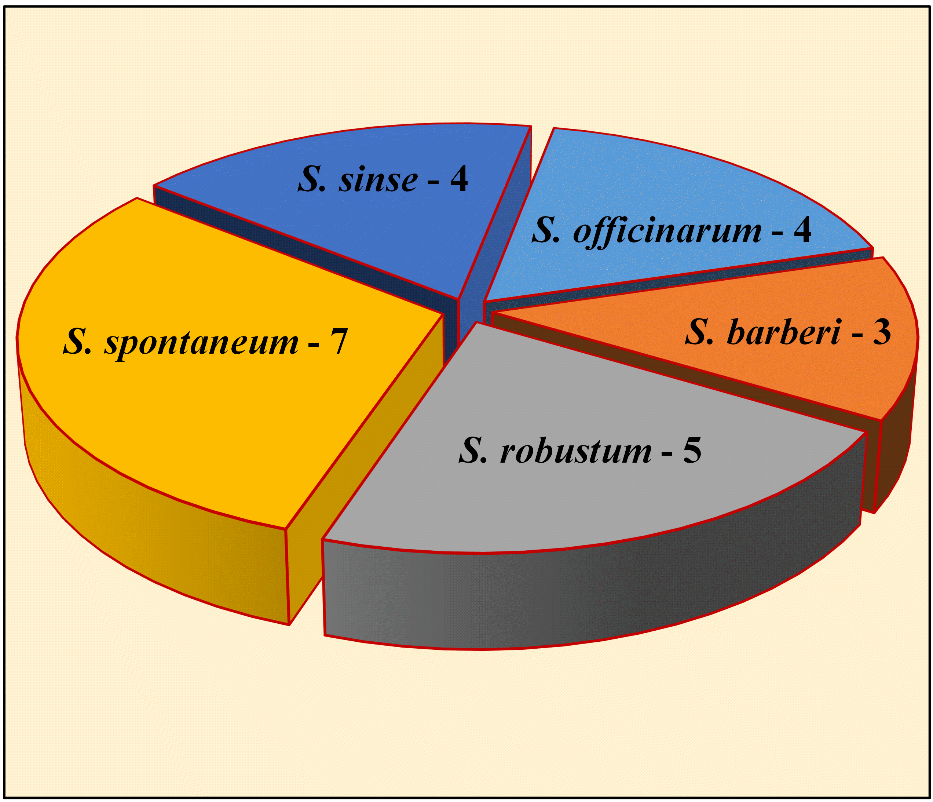


**FIGURE S1** Pie chart displaying the number of selected endophytic *Enterobacter* strains from the root of five different sugarcane species.


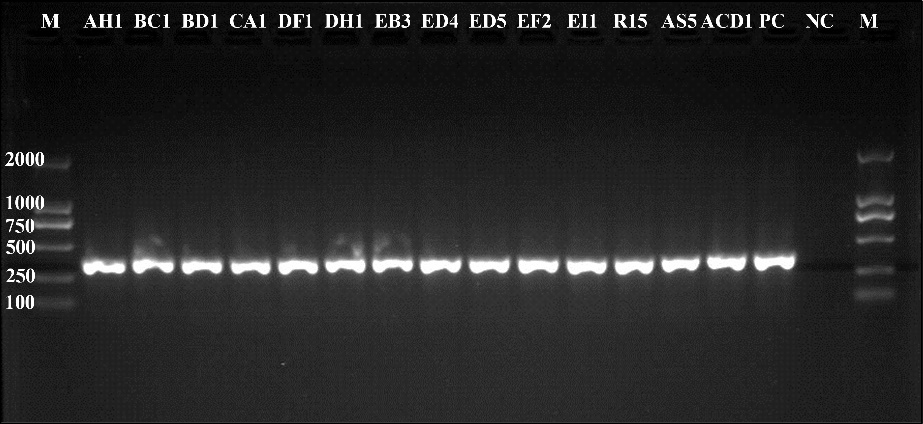


**FIGURE S2** *nifH* gene amplification of positive endophytic *Enterobacter* strains at band size of 360 bp, *Klebsiella verticola* is positive control-P, sterile water is negative control-N, and molecular marker-M from 100 bp- 2 kb (BioFlux) and


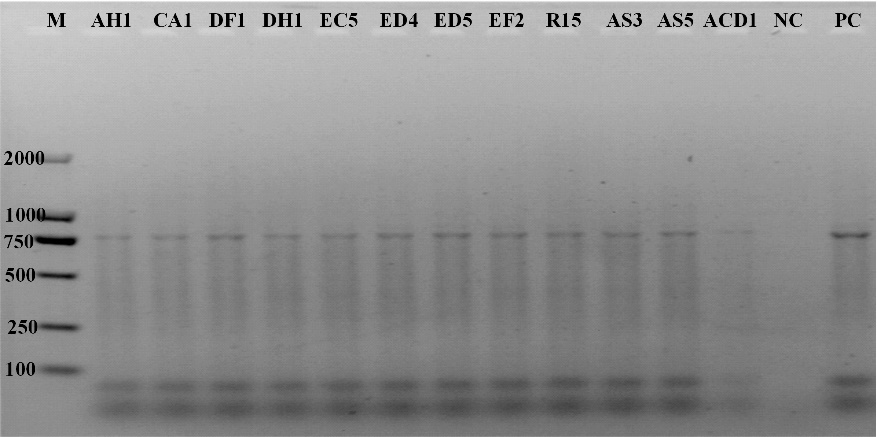


**FIGURE S3** PCR amplification of *acdS* gene in endophytic *Enterobacter* strains at 755bp band size. Molecular marker-M, 100 to 2000 bp (BioFlux), sterile water is negative control-NC, and *P. entomophila* is positive control-PC.


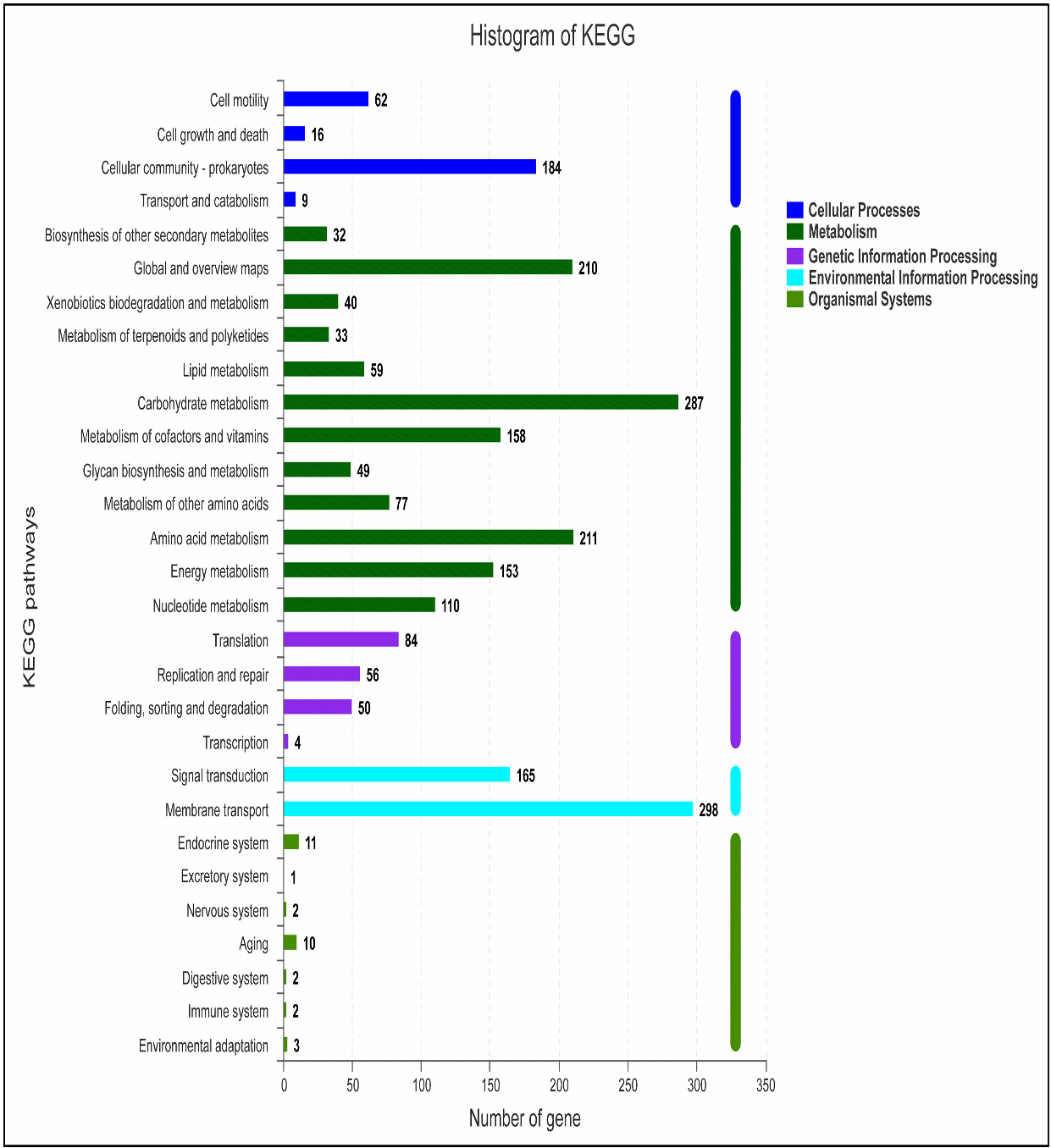


**FIGURE S4** KEGG pathway classification of predicted coding genes of *E. roggenkampii* ED5 genome.


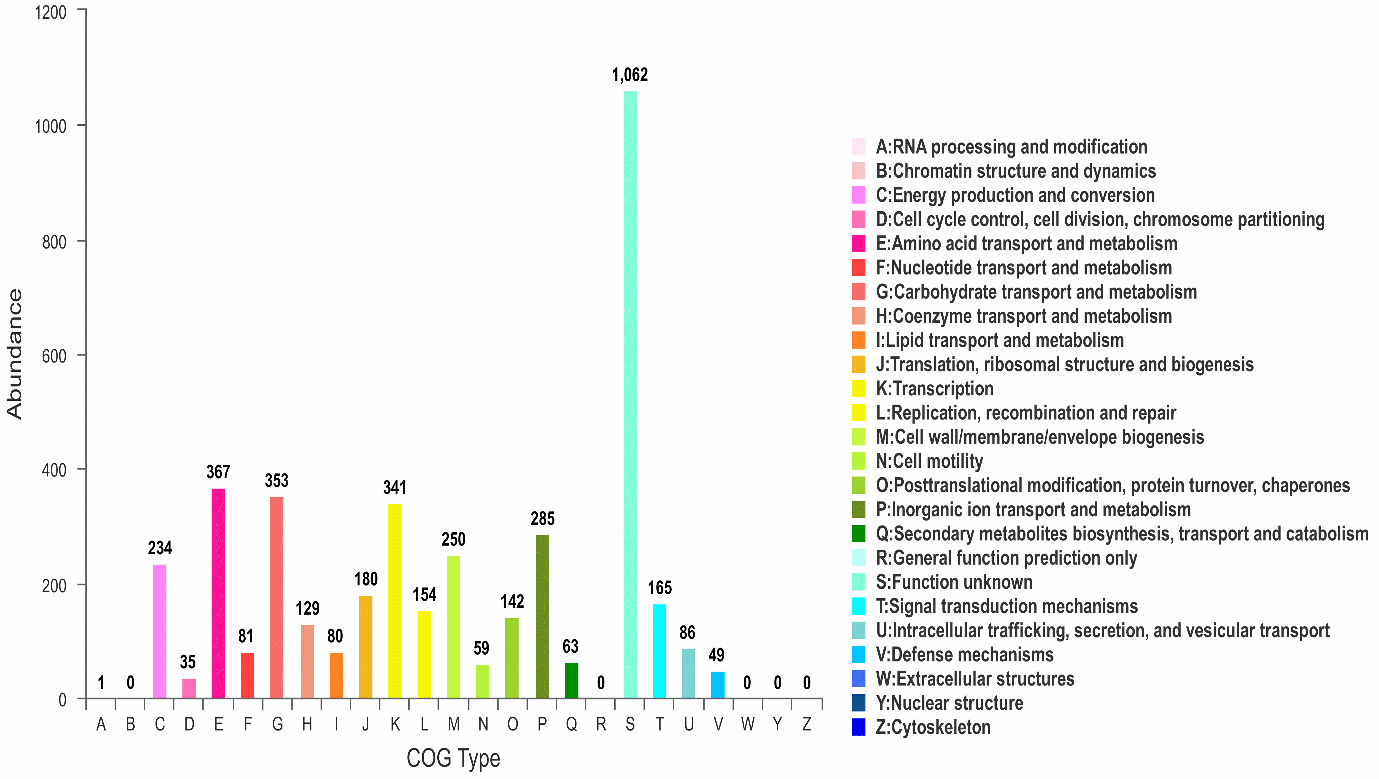


**FIGURE S5** COG functional classification of predicted coding genes of *E. roggenkampii* ED5 genome.


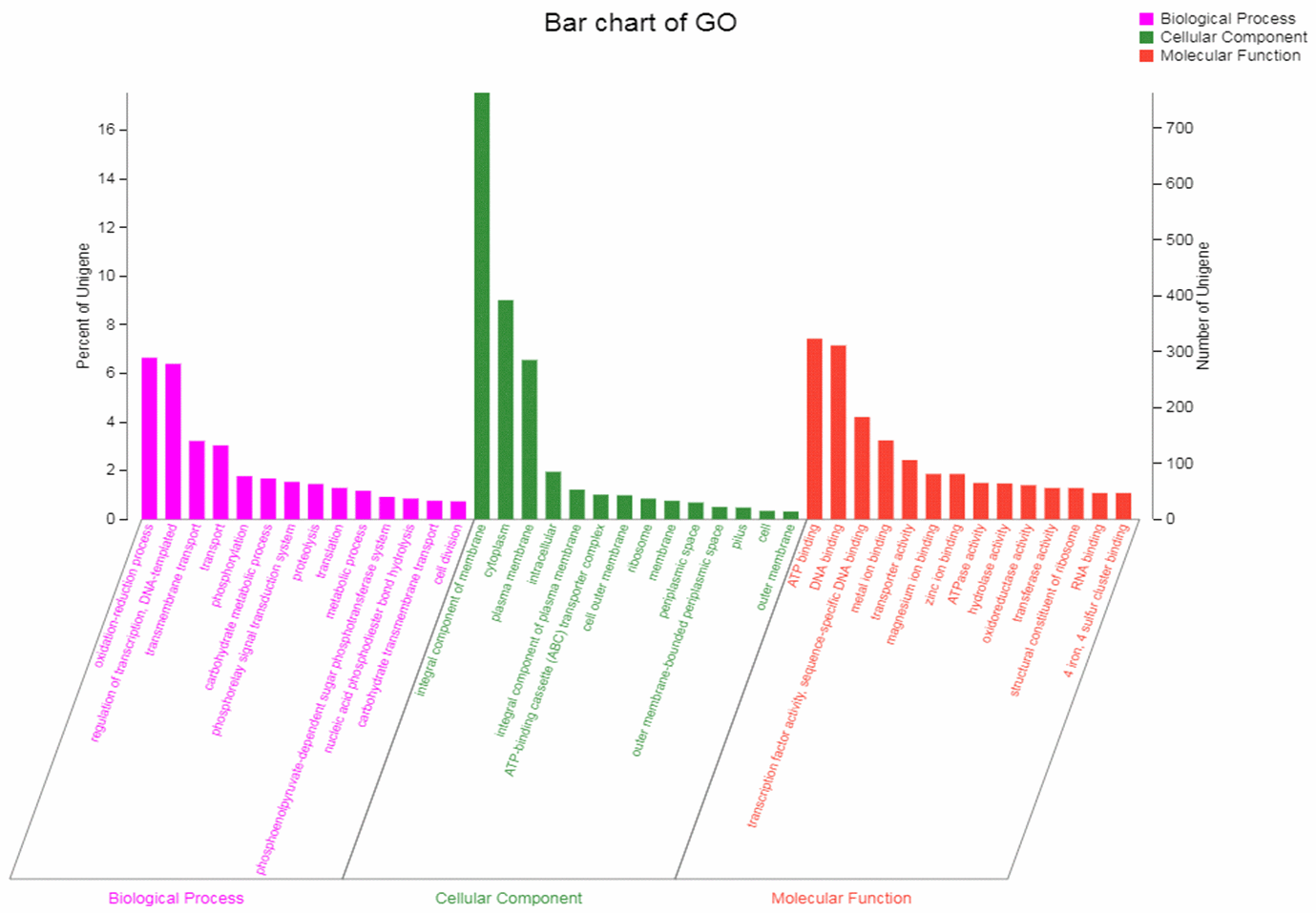


**FIGURE S6** GO analysis of predicted coding genes of *E. roggenkampii* ED5 genome.
